# Influence law of air flows on the risk of secondary oxidation spontaneous combustion of coal

**DOI:** 10.1371/journal.pone.0322637

**Published:** 2025-05-19

**Authors:** Zihao Chai, Baoshan Jia, Ruirui Yu, Wanting Zhao

**Affiliations:** 1 School of Safety Science and Engineering, Liaoning Technical University, Fuxin, Liaoning, China; 2 School of Materials and Chemical Engineering/ School of Safety Engineering, Ningbo University of Technology, Ningbo, Zhejiang, China; 3 College of Mining, Guizhou University, Guiyang, Guizhou, China; Ankara University: Ankara Universitesi, TÜRKIYE

## Abstract

In response to the issue that the self-ignition hazard of coal secondary oxidation under diverse air flow conditions has not been systematically investigated, this study carried out secondary oxidation experiments on lignite under four distinct air flow conditions. By measuring indicators such as the oxygen consumption rate, exothermic intensity, oxygen consumption activation energy, and limiting self-ignition parameters of the coal samples during the experiment, the characteristics of the secondary oxidation hazard of coal under different air flow conditions were deeply explored. The experimental findings indicate that within the air flow range of 25–100 mL/min during the entire oxidation process (40–170°C) and in the first oxidation stage (40–90°C) at 200 mL/min air flow, the oxygen consumption rate and exothermic intensity of the coal samples were significantly higher than those in the primary oxidation process; however, in the second and third oxidation stages (100–170°C) at 200 mL/min air flow, the opposite characteristics were manifested. Additionally, as the air flow decreased, the differences in oxygen consumption rate and exothermic intensity between primary and secondary oxidation gradually diminished. Regarding the oxygen consumption activation energy, the primary oxidation process exhibited lower values in the first and second stages, but in the third stage, the secondary oxidation process at 25 mL/min and 100 mL/min air flow had lower oxygen consumption activation energy, while at 50 mL/min and 200 mL/min air flow, the primary oxidation process had lower oxygen consumption activation energy. The study also discovered that the increase in air flow and the accumulation of oxidation times would significantly enhance the possibility of coal self-ignition and its sustainability.

## 1 Introduction

Despite the Chinese government’s ongoing efforts to reduce coal consumption, China remains one of the world’s largest producers and consumers of coal [1]. Coal spontaneous combustion (CSC) during mining operations not only jeopardizes the safety of underground workers but also leads to substantial economic losses for coal production enterprises. Furthermore, the untreated gases emitted during spontaneous combustion contribute significantly to environmental pollution [2,3]. CSC is a highly complex physicochemical process involving both physical and chemical adsorption, as well as the release of both formal and formless heat [4]. Controlling CSC has become a critical challenge, attracting global scientific attention [5,6]. In China, a considerable number of coal mines face the issue of secondary oxidation in goaf areas, often resulting in uncontrollable fires that necessitate the abandonment of these mines [7]. Secondary oxidation refers to the phenomenon where coal undergoes subsequent combustion after initial oxidation exceeds a critical temperature under favorable ventilation and heat storage conditions [8,9].

Recent studies have shed light on the mechanisms and risks associated with secondary oxidation. For instance, Liu et al. [10] investigated the secondary oxidation of bituminous coal with high variability in quality and found that oxidized coal exhibits enhanced low-temperature oxidation, thereby increasing the risk of spontaneous combustion. Similarly, Liang et al. [11] studied the secondary oxidation of residual coal and demonstrated that, compared to raw coal, residual coal exhibits lower characteristic temperatures and reduced heat absorption during oxidation. Tang et al. [12] focused on lignite and revealed that primary oxidized coal has a higher propensity for spontaneous combustion, particularly during the initial stages of low-temperature oxidation. Wang et al. [13] observed that primary oxidation initially enhances the likelihood of coal spontaneous combustion but later acts as an inhibitory factor under programmed temperature conditions. Zhang et al. [14] further demonstrated that primary oxidation primarily affects the content of functional groups without significantly altering their types, yet it enhances the active structure of coal, making it more susceptible to spontaneous combustion. Guo et al. [15] found that the temperature at which oxidized coal enters the intense oxidation stage is higher than that of raw coal, further complicating the control of CSC.

The presence of air leakage in goaf areas significantly increases the likelihood of coal encountering oxygen, thereby escalating the risk of spontaneous combustion in adjacent coal seams. This poses a severe threat to the safety and productivity of coal mines [16]. Zhang et al. [17] conducted temperature-programmed experiments and demonstrated that variations in airflow significantly influence the occurrence of coal spontaneous combustion. Lei et al. [18] further showed that increasing airflow shifts the region with a high oxygen flow fraction upward, leading to a progressive rise in the internal temperature of coal and an elevated risk of spontaneous combustion. Liu et al. [19] observed that increasing the pre-oxidation process and airflow resulted in higher oxygen consumption and heat release in bituminous coal, further highlighting the critical role of airflow in CSC.

## 2 Experimental materials and procedures

### 2.1 Experimental materials

The lignite used in this experiment was sourced from Shanxi Coal Import and Export Group Zuoyun Donggucheng Coal Industry Co., LTD. The coal sample was crushed to a particle size below 10mm using a hydraulic press with a pressure of 25MPa. Subsequently, the particles were homogeneously mixed through the quaternization method before being loaded into experimental samples.

### 2.2 Experimental methods

For the experiment, a copper canister made of red copper with inner diameter *Φ*=39mm, outer diameter *Φ*=42mm and height *H* = 270mm was chosen as the coal sample canister, and the loading height *h* = 240mm. The coal temperature was measured at the center of the coal sample using a type K thermocouple. The inlet air is heated by a copper inlet pipe, which has an inner diameter of *Φ*=3mm, an outer diameter of *Φ*=4mm, and a length of *L* = 50m. This ensures that the temperature of the coal sample’s inlet air matches the ambient temperature. The experimental setup consisted of four parts: gas supply system, programmed heating system, temperature detection system, and gas chromatography [20].

The coal sample tank was connected to the air supply system, and the experimental air flow was adjusted to facilitate low-temperature primary oxidation with a temperature increase rate of 0.5°C/min. When the temperature of the coal reaches 170°C, heating is ceased, and the gas supply is switched from air to nitrogen. The flow rate of nitrogen is set at 250 mL/min to inhibit coal oxidation and facilitate cooling. When the coal temperature drops to room temperature, re-change the air supply and open the program again to raise the temperature of the coal samples for the second oxidation, the second oxidation process of the heating rate, air flow conditions consistent with the primary oxidation process. During the experiment, the gas was collected at the air outlet at the end of the coal sample tank at intervals of 10°C within the range of 40–170°C, and the components and concentration of the gas were detected by gas chromatograph.

## 3 Experimental results and analysis

### 3.1 Calculation of apparent activation energy of oxygen consumption

**Fig 1:**
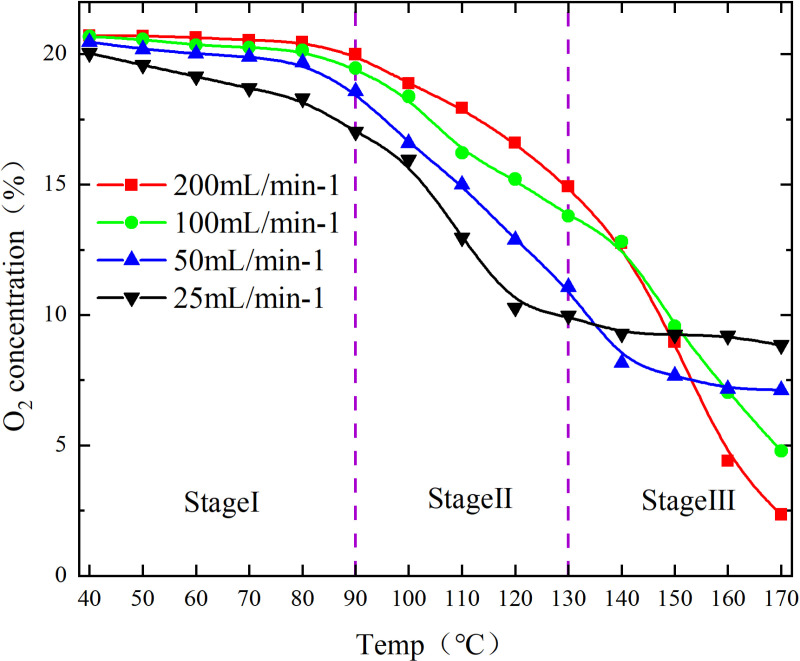
O_2_ concentration under different conditions.

**Fig 2:**
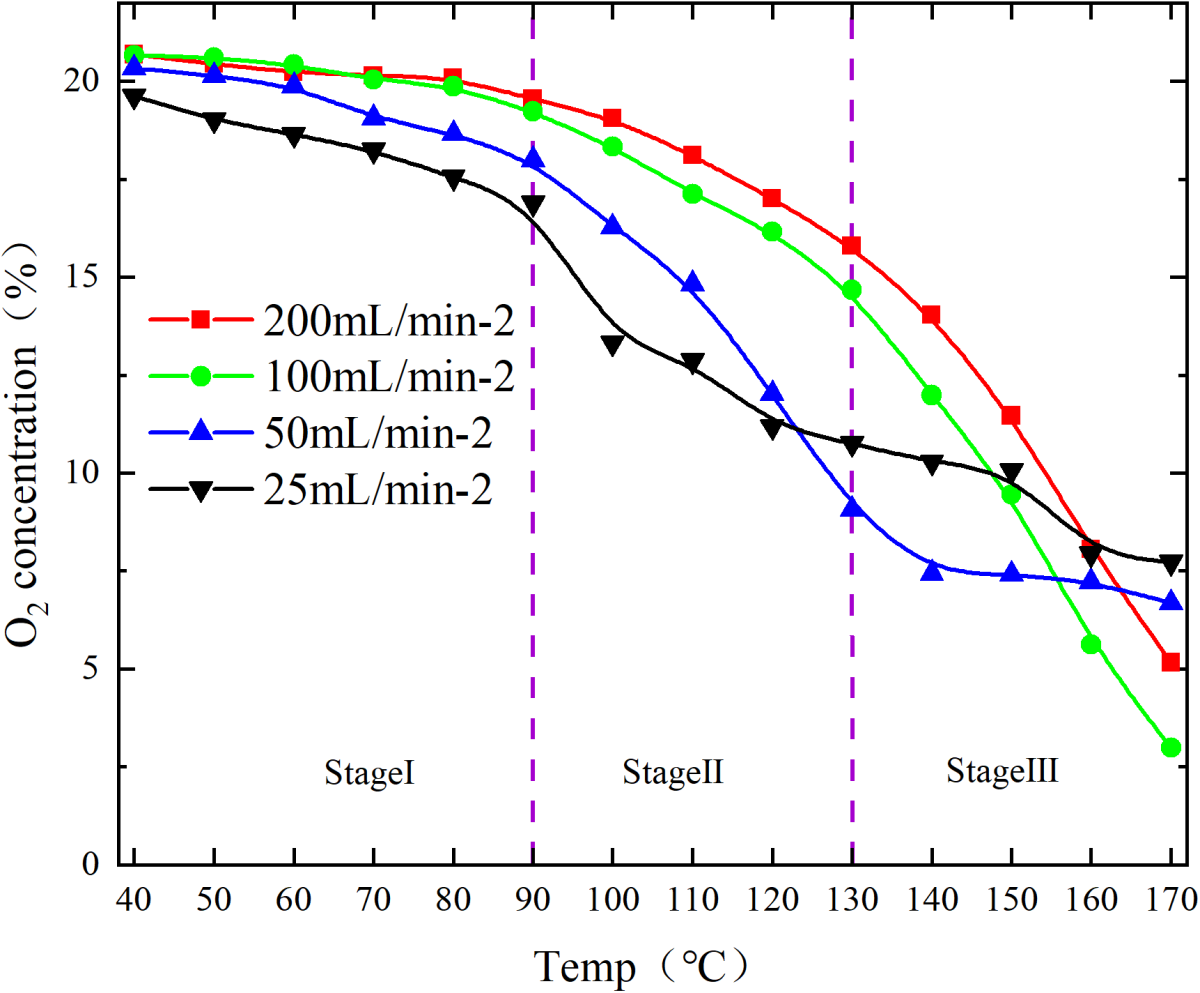
O_2_ concentration under different conditions.

The O_2_ concentration is shown in Figs 1 and 2.

The apparent activation energy of oxygen consumption is a crucial parameter for characterizing the level of oxidation difficulty. A higher value indicates a more challenging coal-oxygen recombination reaction and a reduced risk of spontaneous combustion. The equation for calculating the activation energy of coal is shown in Equation (1)


$$\ln\left[\ln\left(C_{O_{2}}^{in}/C_{O_{2}}^{out}\right)\right]=-\frac{E}{R}\frac{1}{T}+\ln\frac{Av}{Q}$$ (1)

In the equation, $C_{O_{2}}^{in}$ represents the actual inlet oxygen concentration, mol/ cm^3^; $C_{O_{2}}^{out}$ represents actual outlet oxygen concentration, mol/ cm^3^;A represents pre-factor, s^-1^; E represents apparent activation energy, J/mol; R represents gas constant, 8.314J/(mol·k); T represents thermodynamic temperature, K; Q represents air delivery flow, cm^3^/s; v represents flow of coal sample, cm^3^.

Let 1/T=x,$\ln\left[\ln\left(C_{O_{2}}^{in}/C_{O_{2}}^{out}\right)\right]=y$,then$y=-\frac{E}{R}x+\ln\frac{Av}{Q}$. Let$-\frac{E}{R}=\text{a}$,$\ln\frac{Av}{Q}=b$,then y=ax+b,followed by E=−aR.

**Fig 3:**
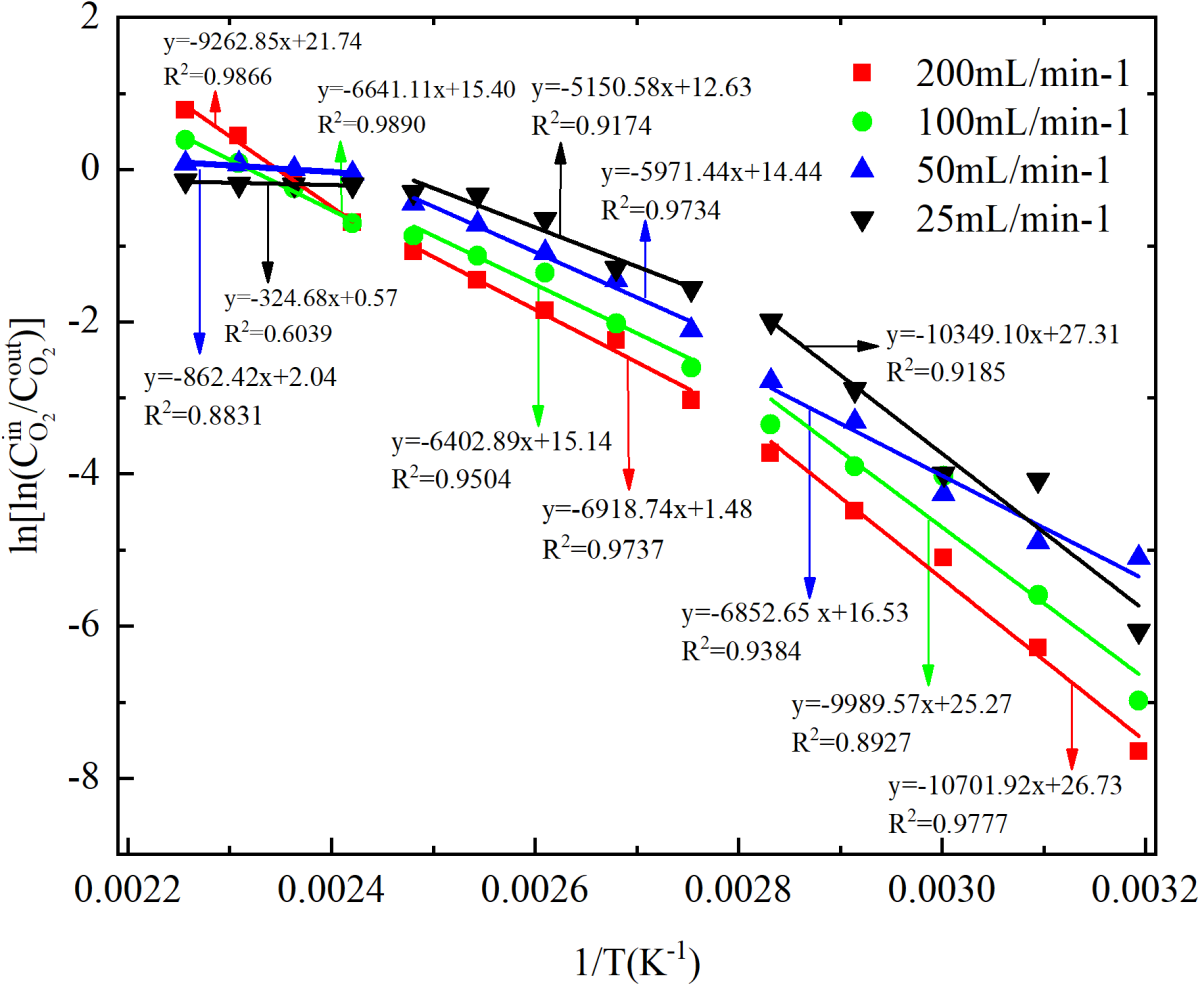
Fitting of coal samples $\ln\left[\ln\left(C_{O_{2}}^{in}/C_{O_{2}}^{out}\right)\right]$ to 1/T under different conditions.

**Fig 4:**
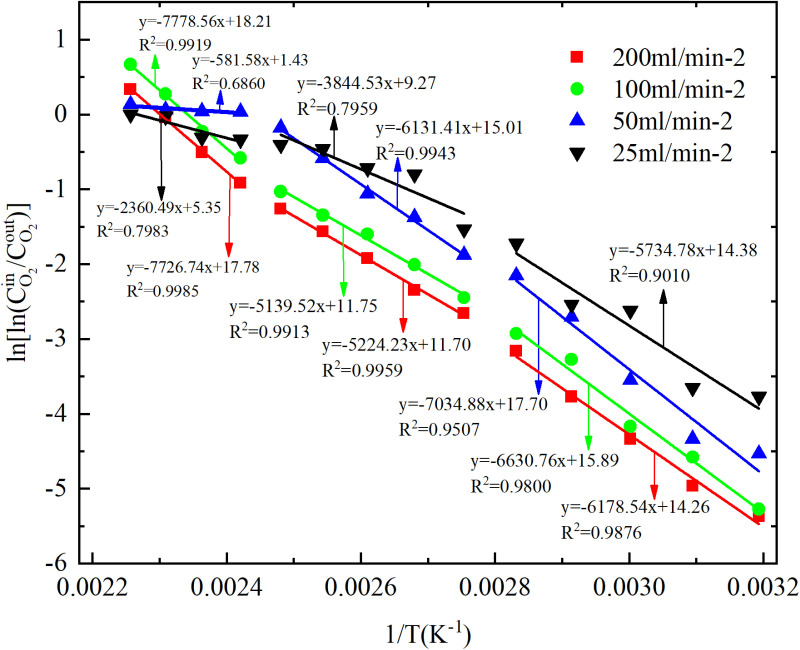
Fitting of coal samples $\ln\left[\ln\left(C_{O_{2}}^{in}/C_{O_{2}}^{out}\right)\right]$ to 1/T under different conditions.

**Fig 5:**
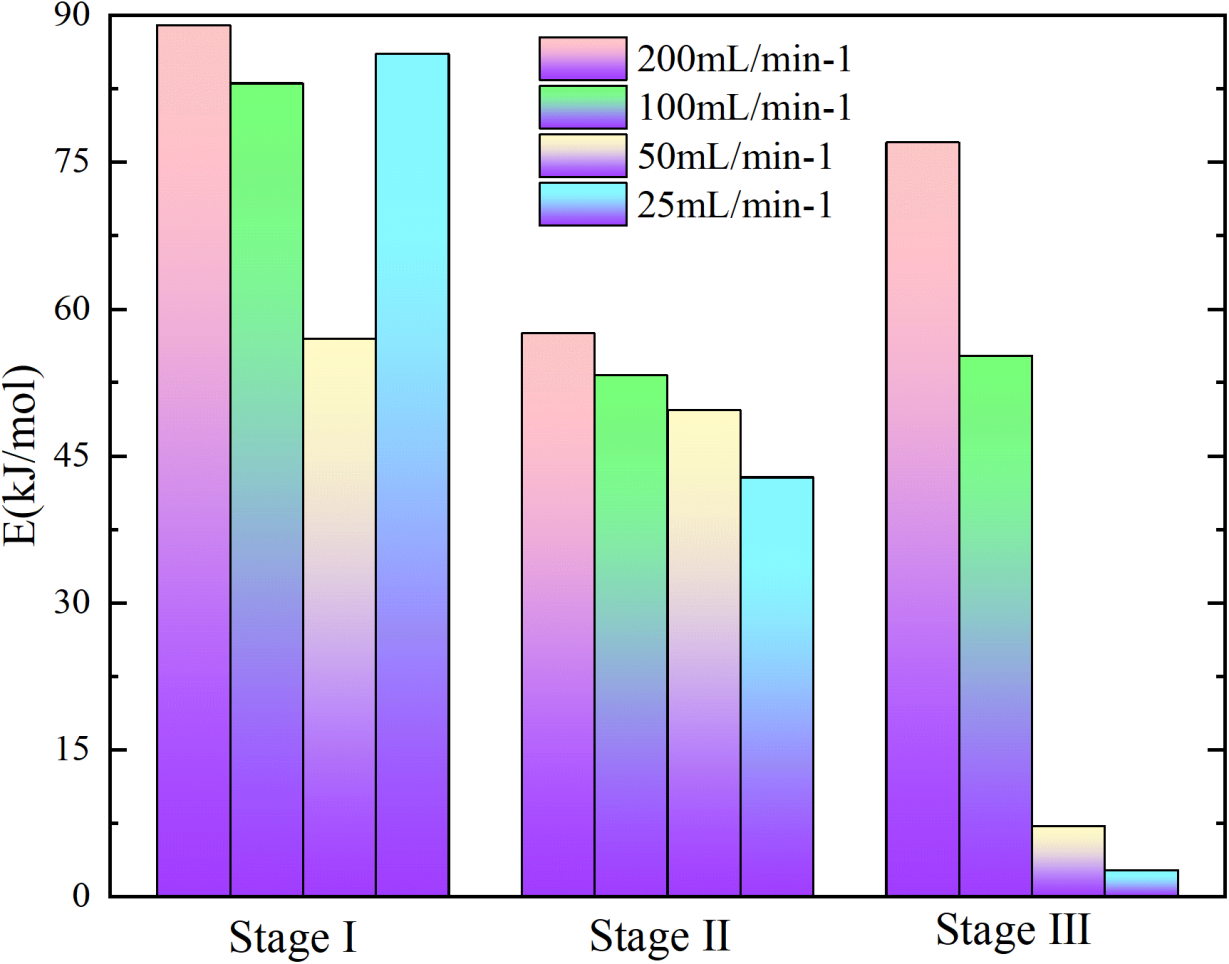
Apparent activation energy of coal samples under different experimental conditions.

**Fig 6:**
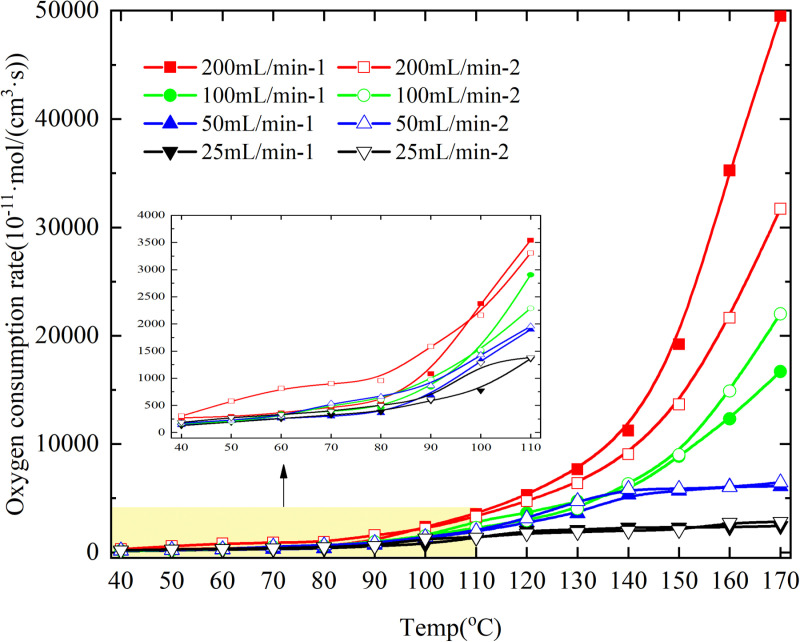
Apparent activation energy of coal samples under different experimental conditions.

The low-temperature oxidation (40–170°C) process of experimental coal samples can be categorized into three stages, based on the relationship between oxygen concentration and coal temperature during CSC, as depicted in Figs 1 and 2. The stage Ⅰ involves coal temperatures ranging from 40 to 80°C, during which the oxygen consumption is relatively sluggish, and the concentration changes exhibit minimal variations; The coal temperature reaches 90–130°C in the stage Ⅱ, leading to an acceleration in oxygen consumption. As a result, the oxygen concentration rapidly declines and the disparity between each air flow increases; The stage Ⅲ is characterized by a coal temperature range of 140–170°C. Within this temperature range, the rate of oxygen consumption accelerates, leading to a significant decrease in oxygen concentration and an increase in the disparity between air flow. A linear fit with 1/T as the horizontal coordinate and $\ln\left[\ln\left(C_{O_{2}}^{in}/C_{O_{2}}^{out}\right)\right]$ as the vertical coordinate was performed to obtain the y=ax+b and R^2^ for each segment as shown in Figs 3 and 4, followed by the apparent activation energy as shown in Figs 5 and 6.

In the process of primary and secondary oxidation, the coal samples exhibited a decrease in the secondary oxidation stage I by 42.27%, 33.62%, and 44.59% respectively, compared to the primary oxidation, under experimental air flows of 200mL/min, 100mL/min, and 25mL/min. Additionally, at an air flow of 50mL/min, the secondary oxidation stage I of the coal sample was found to be 2.66% higher than that observed during primary oxidation. The coal sample exhibited a reduction of 24.49%, 19.73%, and 25.36% in the secondary oxidation stage II compared to the primary oxidation when subjected to experimental air flows of 200mL/min, 100mL/min, and 25mL/min, respectively. Additionally, there was a slight increase of 2.68% in the secondary oxidation stage II for the coal sample under an experimental air flow of 50mL/min compared to the primary oxidation. Under the experimental air flows of 200mL/min and 50mL/min, the secondary oxidation stage III of the coal sample exhibits a reduction of 16.58% and 32.56%, respectively, compared to the primary oxidation stage. Conversely, at air flows of 100mL/min and 25mL/min, the secondary oxidation stage III shows an increase of 17.13% and a remarkable surge by 627.02%, respectively, in comparison with the primary oxidation stage. The results demonstrate that the primary oxidation process can decrease the apparent activation energy of coal during stages Ⅰ and Ⅱ, lower the apparent activation energy of coal samples at air flow rates of 200mL/min and 50mL/min during stage Ⅲ, and increase the apparent activation energy of coal samples at air flow rates of 100mL/min and 25mL/min.

### 3.2 The difference of the change rule of CO_x_ generation rate

CO and CO_2_ generation rate can characterize the intensity of coal-oxygen complexation. Based on the experimentally determined O_2_, CO, and CO_2_ concentrations, Equation (2) and Equation (3) were calculated in conjunction with the CO and CO_2_ generation rates:


$$V_{CO}(T)=\frac{V_{O_{2}}(T)C_{CO}^{out}}{C_{O_{2}}^{in}\left(1-e^{-\frac{V_{O_{2}}(T)vn}{QC_{O2}^{in}}}\right)}$$ (2)

$$V_{CO_{2}}(T)=\frac{V_{O_{2}}(T)C_{CO_{2}}^{out}}{C_{O_{2}}^{in}\left(1-e^{-\frac{V_{O_{2}}(T)vn}{QC_{O2}^{in}}}\right)}$$ (3)

In equations, $V_{CO}(T)$ represents the rate of production of carbon monoxide, mol/(cm^3^·s); $V_{CO_{2}}(T)$ represents the rate of production of carbon dioxide, mol/(cm^3^·s);$C_{CO}^{out}$ represents actual outlet carbon monoxide concentration, mol/cm^3^; $C_{CO_{2}}^{out}$ represents actual outlet carbon dioxide concentration, mol/ cm^3^.

**Fig 7:**
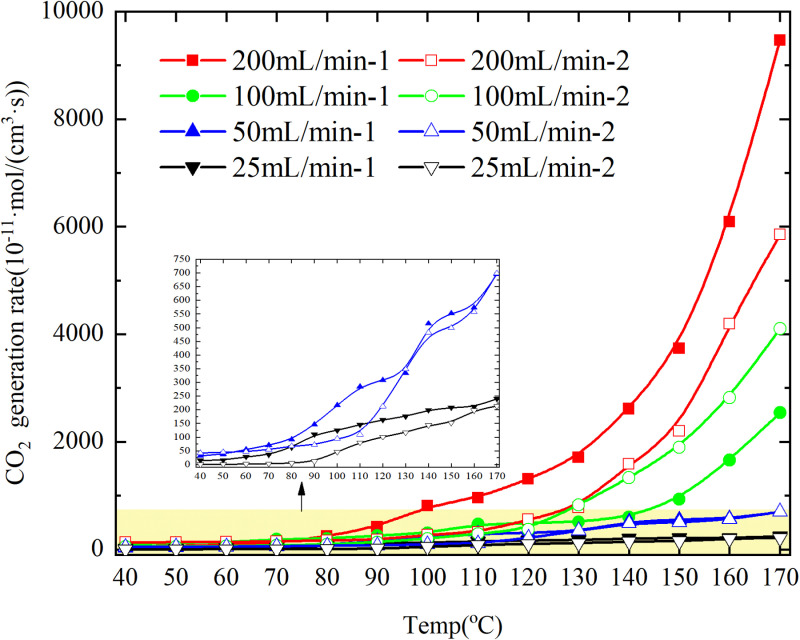
CO_2_ &CO generation rate of primary and secondary oxidation of coal under different air flows.

**Fig 8:**
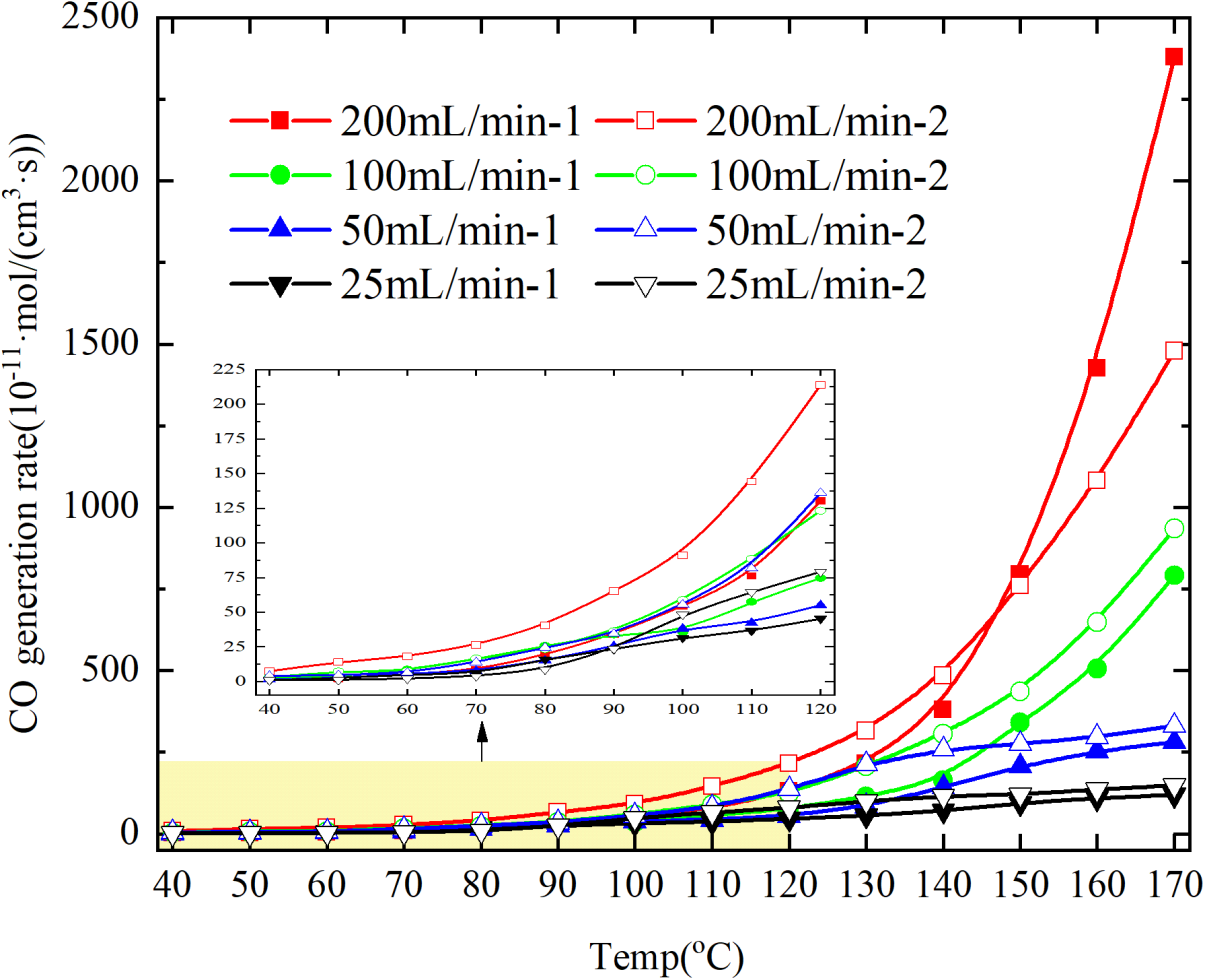
CO_2_ &CO generation rate of primary and secondary oxidation of coal under different air flows.

**Fig 9:**
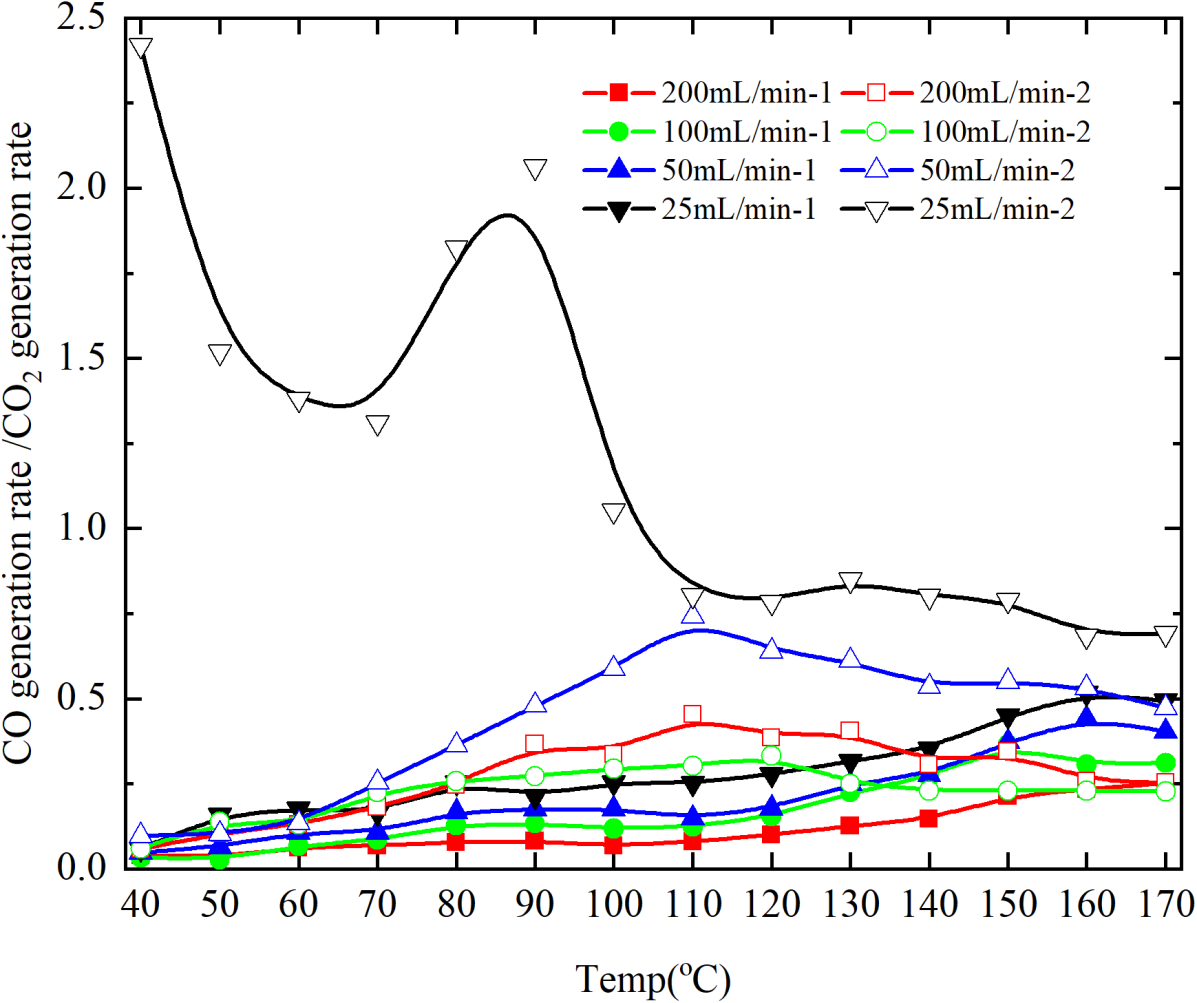
CO/CO_2_ generation rate of primary and secondary oxidation of coal under different air flows.

The rates of CO and CO_2_ production in the coal samples during the programmed warming are shown in Figs 7 and 8 and CO generation rate/CO_2_ generation rate is shown in Fig 9.

As depicted in Fig 7, the CO_2_ generation rate of coal under an experimental air flow of 100mL/min is higher than that of primary oxidation during secondary oxidation. However, for other experimental air flow conditions, the CO_2_ generation rate of coal is generally higher during primary oxidation compared to secondary oxidation. Furthermore, the disparity in CO_2_ generation rates between primary and secondary oxidation increases with escalating air flows.

The CO generation rate of primary oxidation is higher than that of secondary oxidation for coal with an experimental air flow of 200mL/min at a temperature of 140°C, as depicted in Fig 8. However, for other air flows, the CO generation rate of primary oxidation is lower than that of secondary oxidation.

As depicted in Fig 9, except for the coal sample exhibiting an experimental air flow of 100mL/min and a coal temperature of 130°C, the ratio of CO/CO_2_ generation rate during primary oxidation surpasses that observed during secondary oxidation. Conversely, for other coal samples under varying air flow conditions, the ratio of CO/CO_2_ generation rate is lower than that observed during secondary oxidation. The results show that the coal samples with the experimental air flows of 200mL/min, 50mL/min and 25mL/min are more incomplete in the whole process of secondary oxidation, and the coal samples with the experimental air flow of 100mL/min are more incomplete in the coal temperature range of 40–130°C.

### 3.3 Calculation of oxygen consumption rate

**Fig 10:**
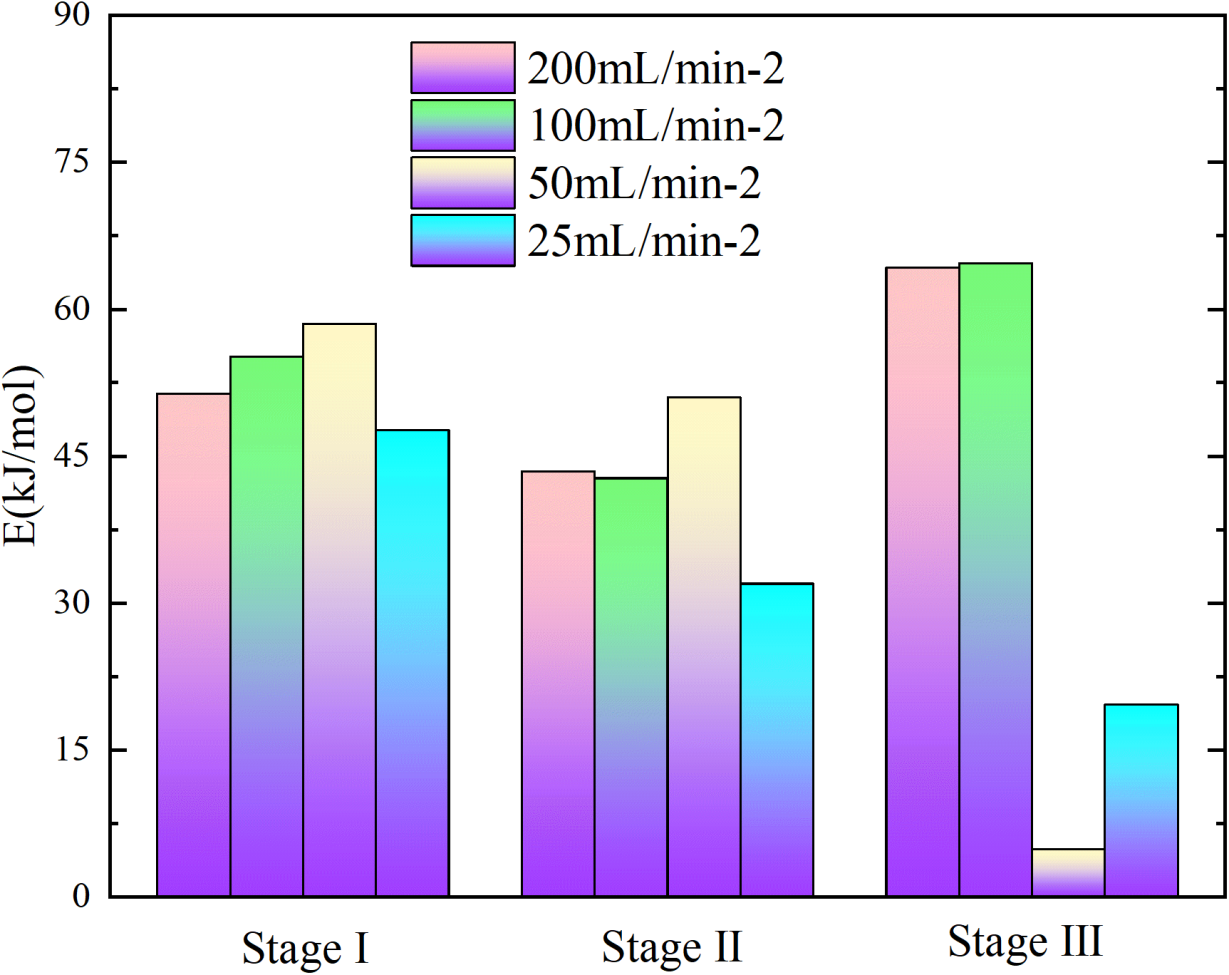
Oxygen consumption rate under different experimental conditions.


The oxygen consumption rate of the coal samples was calculated according to the oxygen consumption rate Equation (4), and its variation with temperature is shown in Fig 10.

$$V_{O_{2}}(T)=\frac{QC_{O_{2}}^{in}}{v}ln\frac{C_{O_{2}}^{out}}{C_{O_{2}}^{in}}$$ (4)

In the equation, $V_{O_{2}}(T)$ represents oxygen consumption rate, mol/(cm^3^·s); Q represents air flow, cm^3^/s; v represents flow of coal samples, cm^3^; $C_{O_{2}}^{in}$ represents actual inlet oxygen concentration, mol/ cm^3^; $C_{O_{2}}^{out}$ represents actual outlet oxygen concentration, mol/ cm^3^.

The oxygen consumption rate difference at the experimental temperature point increases with the increase of air flow, as depicted in Fig 10, during both primary and secondary oxidation processes. The comparison between primary and secondary oxidation reveals that the coal sample exhibits a higher rate of oxygen consumption during secondary oxidation at coal temperatures ranging from 40–90°C under an experimental air flow of 200mL/min, while the opposite trend is observed when the coal temperature exceeds 90°C. This is attributed to the presence of a significant number of active functional groups following the primary oxidation of coal, which can undergo secondary oxidation within the temperature range of 40–90°C. Simultaneously, a considerable number of functional groups undergo reaction with an adequate amount of oxygen molecules during the primary oxidation process of coal samples, leading to a reduction in the overall count of functional groups capable of participating in secondary oxidation. Consequently, within the temperature range of 90–170°C, the rate at which oxygen is consumed during secondary oxidation is diminished.

The oxygen consumption rate of the coal sample during the secondary oxidation process, under an experimental air flow of 100mL/min, exhibits a higher value compared to that observed solely during the secondary oxidation within the temperature range of 110–130°C. This finding leads to the conclusion that at an experimental air flow of 100mL/min, the oxygen consumption rate of the coal sample is enhanced during its secondary oxidation process. The oxygen consumption rate of the coal sample under an experimental air flow of 50mL/min surpasses that observed during primary oxidation throughout the entire process, albeit with a marginal disparity in oxygen consumption rates between the two distinct oxidation processes. The oxygen consumption rate of primary oxidation is only higher at coal temperatures between 120–150°C under an experimental air flow of 25mL/min, with a maximum difference of 286.59 × 10^-11^mol/(cm^3^·s) between primary and secondary oxidation. It can be concluded that the oxygen consumption rate during secondary oxidation is higher under an experimental air flow of 25mL/min, and the maximum difference in oxygen consumption rate between the two processes is smaller than that observed under an experimental air flow of 50mL/min. he results demonstrate that when the experimental air flow is less than or equal to 100 mL/min, the oxygen consumption rate of secondary coal oxidation surpasses that of primary oxidation. This can be attributed to the presence of a substantial number of active functional groups resulting from heat-induced reactions after primary oxidation. However, due to limited air flow, the overall availability of oxygen molecules for reaction with these active functional groups remains relatively low, leading to weaker intensity in the reaction process. Consequently, there is no significant increase in the oxygen consumption rate during secondary oxidation compared to primary oxidation; moreover, as the air flow decreases, the curves depicting oxygen consumption rates for both primary and secondary oxidations tend to converge.

### 3.4 Calculation and analysis of the exothermic intensity

The heat released by coal-oxygen composite action is the main heat source of spontaneous combustion of coal, and the exothermic intensity is its important index. The upper limit of exothermic intensity is calculated by the Equation (5), Equation (6) and Equation (7).


$$q_{\max}(T)=\frac{V_{CO}(T)}{V_{CO}(T)+V_{CO_{2}}(T)}V_{O_{2}}(Tcdot\Delta H^{CO}+\frac{V_{CO_{2}}(T)}{V_{CO}(T)+V_{CO_{2}}(T)}V_{O_{2}}(T)\cdot \Delta H^{CO_{2}}$$ (5)

$${\text{q}}_{\min}(T)=\Delta H^{\alpha }[V_{O_{2}}(T)-V_{CO}(T)-V_{CO_{2}}(T)]+\Delta H^{CO}V_{CO}(T)+\Delta H^{CO_{2}}V_{CO_{2}}(T)$$ (6)

$$\text{q}(T)=\frac{1}{2}[{\text{q}}_{\min}(T)+{\text{q}}_{\max}(T)]$$ (7)

In the equation, $q_{\max}(T)$, ${\text{q}}_{\min}(T)$, q(T) represents upper limit of exothermic intensity, lower limit of exothermic intensity, exothermic intensity, respectively, kJ/(cm^3^·s); $\Delta H^{CO}$ represents average heat of reaction of CO, $\Delta H^{CO}$ = 311.9kJ/mol; $\Delta H^{CO_{2}}$ represents average heat of reaction of CO_2_, $\Delta H^{CO_{2}}$ = 446.7 kJ/mol.

**Fig 11:**
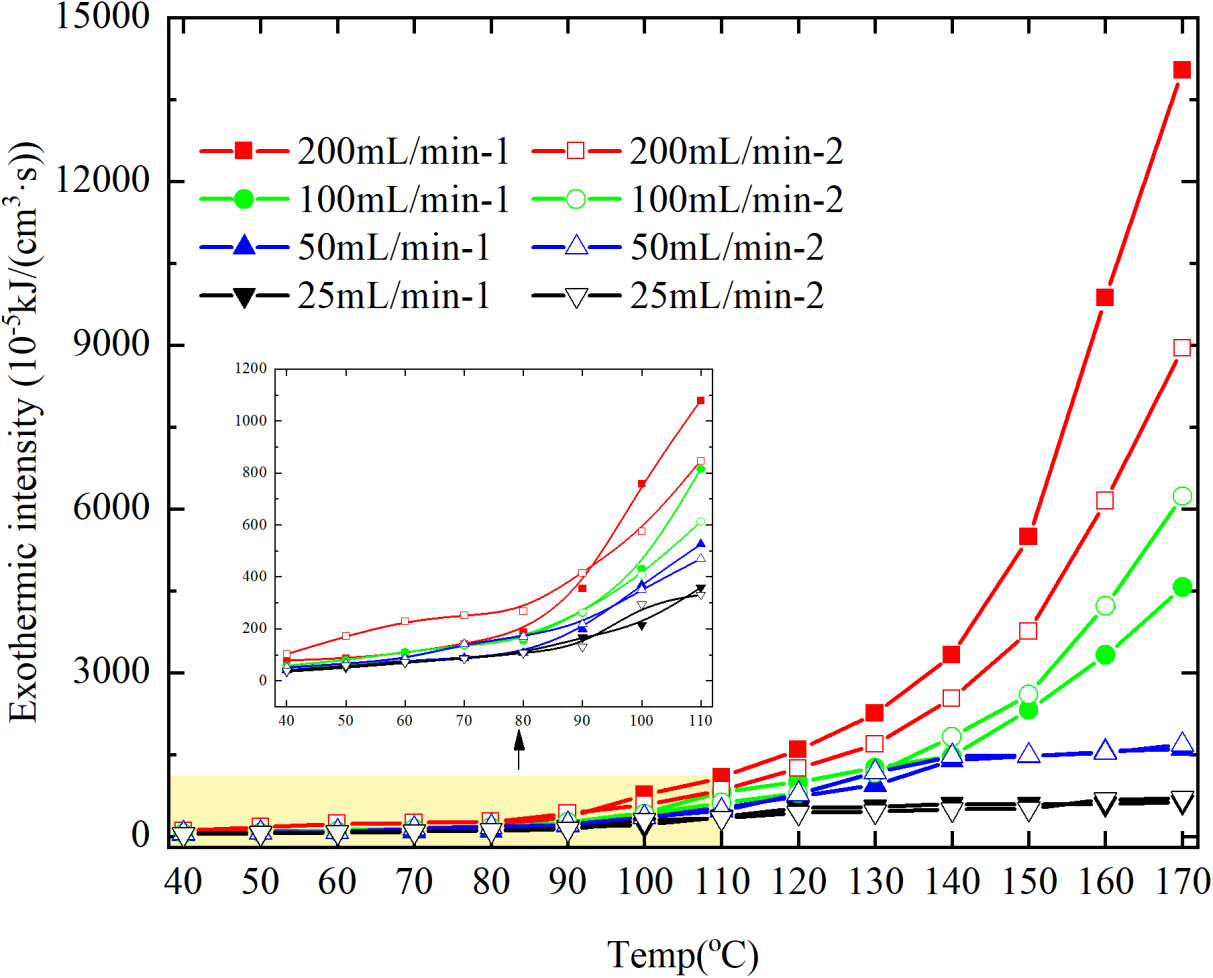
Exothermic intensity of coal samples under different conditions.

The exothermic intensity of coal samples is shown in Fig 11.

As depicted in Fig 11, the disparity in exothermic intensity at the experimental temperature point increases with escalating air flow during both primary and secondary oxidation processes of coal. The coal sample exhibits a higher exothermic intensity of secondary oxidation in the temperature range of 40–90°C under an experimental air flow of 200mL/min, compared to primary and secondary oxidation, whereas the opposite trend is observed beyond 90°C. This observation aligns with the oxygen consumption rate law, indicating a greater activation of functional groups during the secondary oxidation process compared to the primary oxidation process at coal temperatures ranging from 40–90°C, resulting in enhanced exothermic intensity. However, when the coal temperature exceeds 90°C, there is a reduction in the number of participating functional groups during secondary oxidation, leading to a decrease in exothermic intensity relative to primary oxidation. For coal samples with an air flow of 100mL/min, the exothermic intensity during primary oxidation exceeds that of secondary oxidation only within the temperature range of 110–130°C. However, for coal samples with an air flow of 50mL/min, the exothermic intensity during secondary oxidation surpasses that of primary oxidation throughout total oxidation. Under the condition of an air flow of 25mL/min, the exothermic intensity during primary oxidation is greater than that of secondary oxidation only within the coal temperature range of 120–150°C, with a maximum difference observed at 99.61 × 10^-5^ kJ/(cm^3^·s). Therefore, it can be inferred that the exothermic intensity of coal during the secondary oxidation process is higher when the experimental air flow is less than or equal to 100mL/min, which aligns with the observed trend in oxygen consumption rate.

## 4 Calculation and analysis of limiting parameters

CSC is a result of the joint action of intrinsic and extrinsic factors of coal, and CSC may occur when both intrinsic and extrinsic factors fulfill the conditions. The limit value of external conditions under which coal can spontaneous combustion is called CSC limit parameter, which mainly includes minimal thickness of residual coal, limit oxygen concentration and lower limit air leakage intensity. The calculation equations for the minimal thickness of residual coal, the lower limit oxygen concentration, and the maximal air leakage intensity are shown in Equation (6), Equation (7), and Equation (8), respectively.


$$h_{\min}=\frac{\rho_{g}C_{g}q(T-T_{y})+\sqrt{{\left(\rho_{g}C_{g}q\right)}^{2}{\left(T-T_{y}\right)}^{2}+8\lambda_{e}Q(T)(T-T_{y})}}{Q(T)}$$ (6)

$$C_{\min}^{O_{2}}=\frac{C_{e}^{O_{2}}}{Q(T)}\left[\frac{8\lambda_{e}(T-T_{y})}{h^{2}}+\rho_{g}C_{g}q\frac{2(T-T_{y})}{h}\right]$$ (7)

$$q_{\max}=\frac{hQ(T)}{2\rho_{g}C_{g}(T-T_{y})}-\frac{4\lambda_{e}}{h\rho_{g}C_{g}}$$ (8)

In equations, $h_{\min}$ represents minimum floating coal thickness, cm; $\rho_{g}$ represents air density, kg/m^3^; $C_{g}$ represents specific heat capacity of air, J/(kg·K); q represent air leakage intensity, cm/s; $T_{y}$ represents temperature of the coal body enclosure, °C; T represents the air flow temperature, °C; $\lambda_{e}$ represents equivalent thermal conductivity of the loose coal body, J/(cm·s·K); Q(T) represents exothermic intensity of the loose coal body at temperature T, J/(cm^3^·s); $C_{\min}^{O_{2}}$ represents low limit oxygen flow fraction, %; $C_{e}^{O_{2}}$ represents oxygen concentration in the air, %; h represents thickness of the loose coal body, cm; $q_{\max}$ represents maximal air leakage intensity, cm/s.

**Fig 12:**
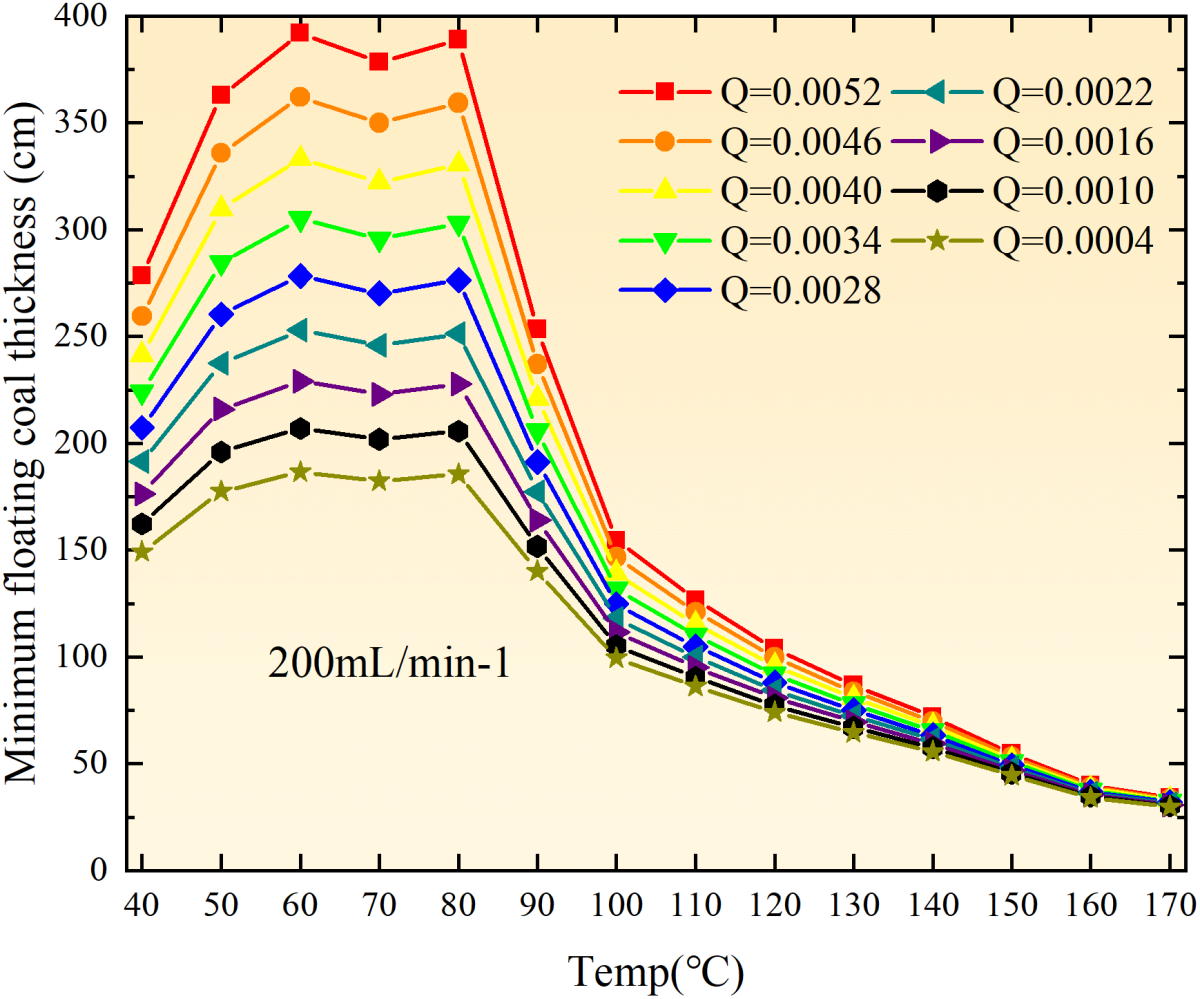
The minimum thickness of float coal under different air leakage intensities.

**Fig 13:**
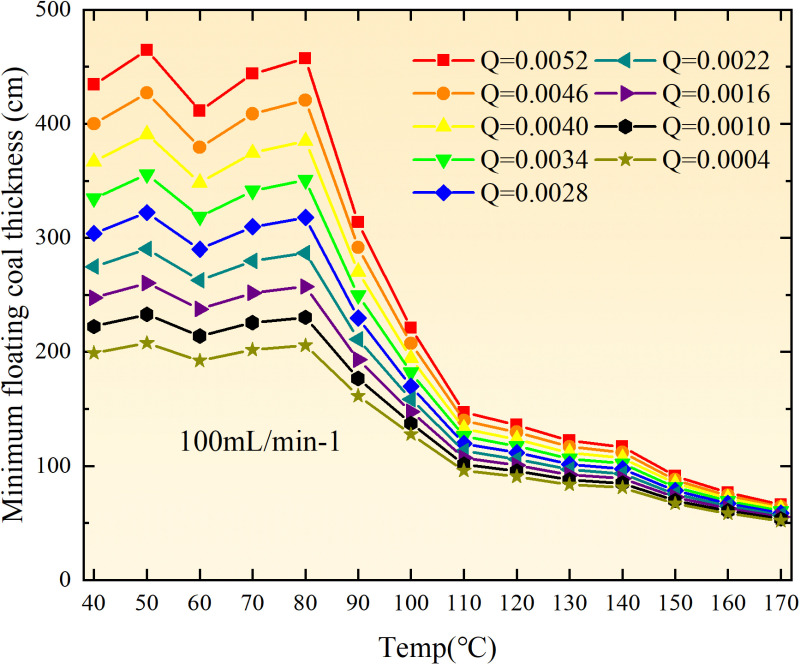
The minimum thickness of float coal under different air leakage intensities.

**Fig 14:**
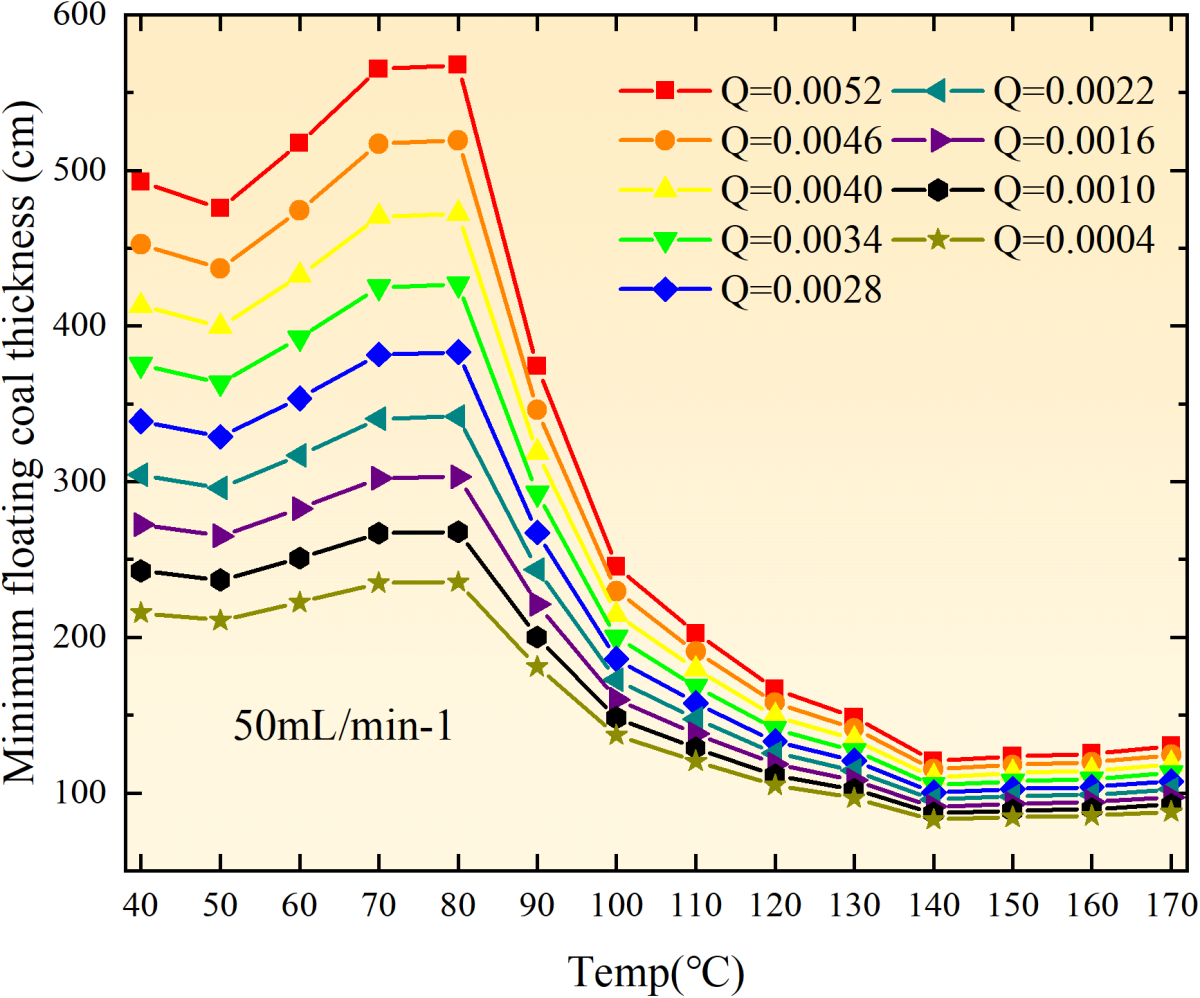
The minimum thickness of float coal under different air leakage intensities.

**Fig 15:**
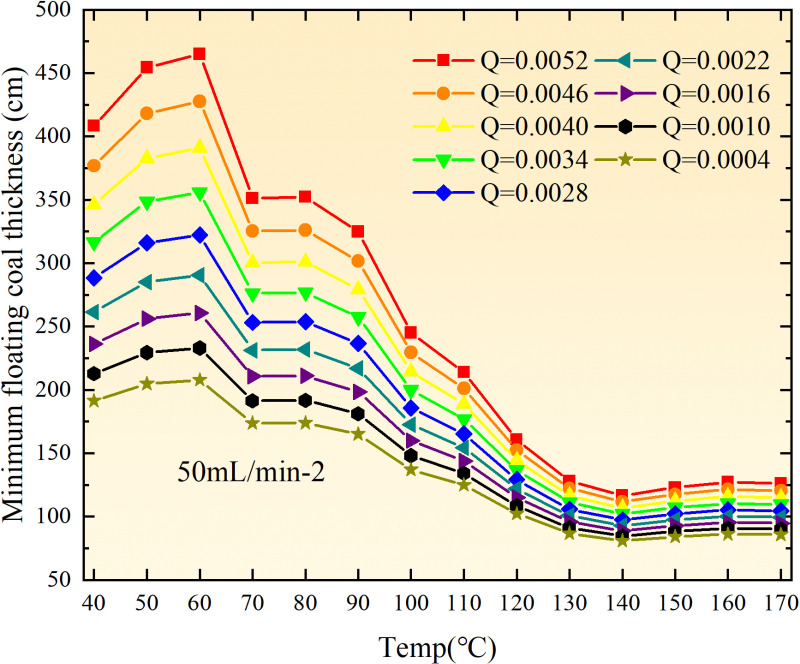
The minimum thickness of float coal under different air leakage intensities.

**Fig 16:**
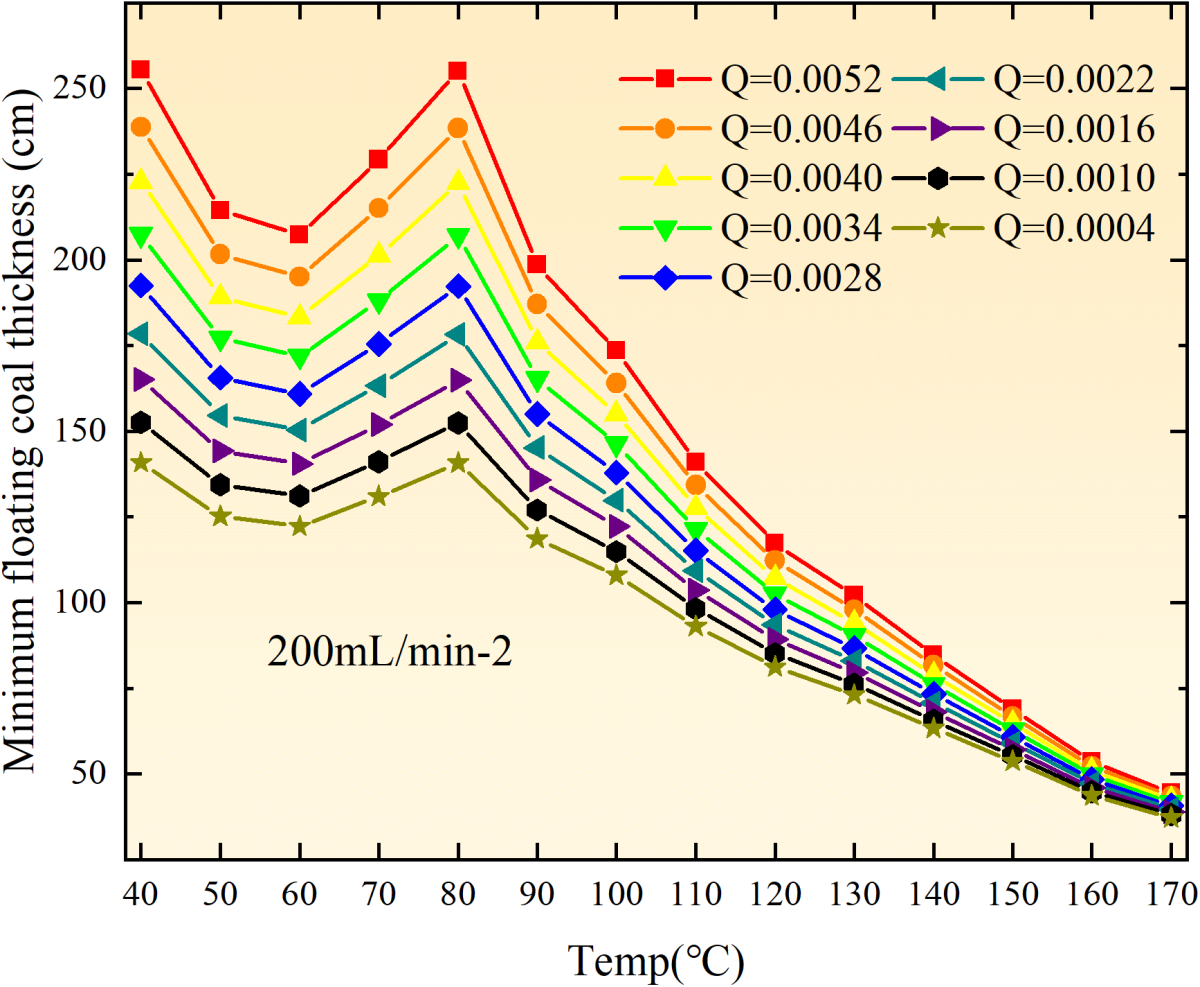
The minimum thickness of float coal under different air leakage intensities.

**Fig 17:**
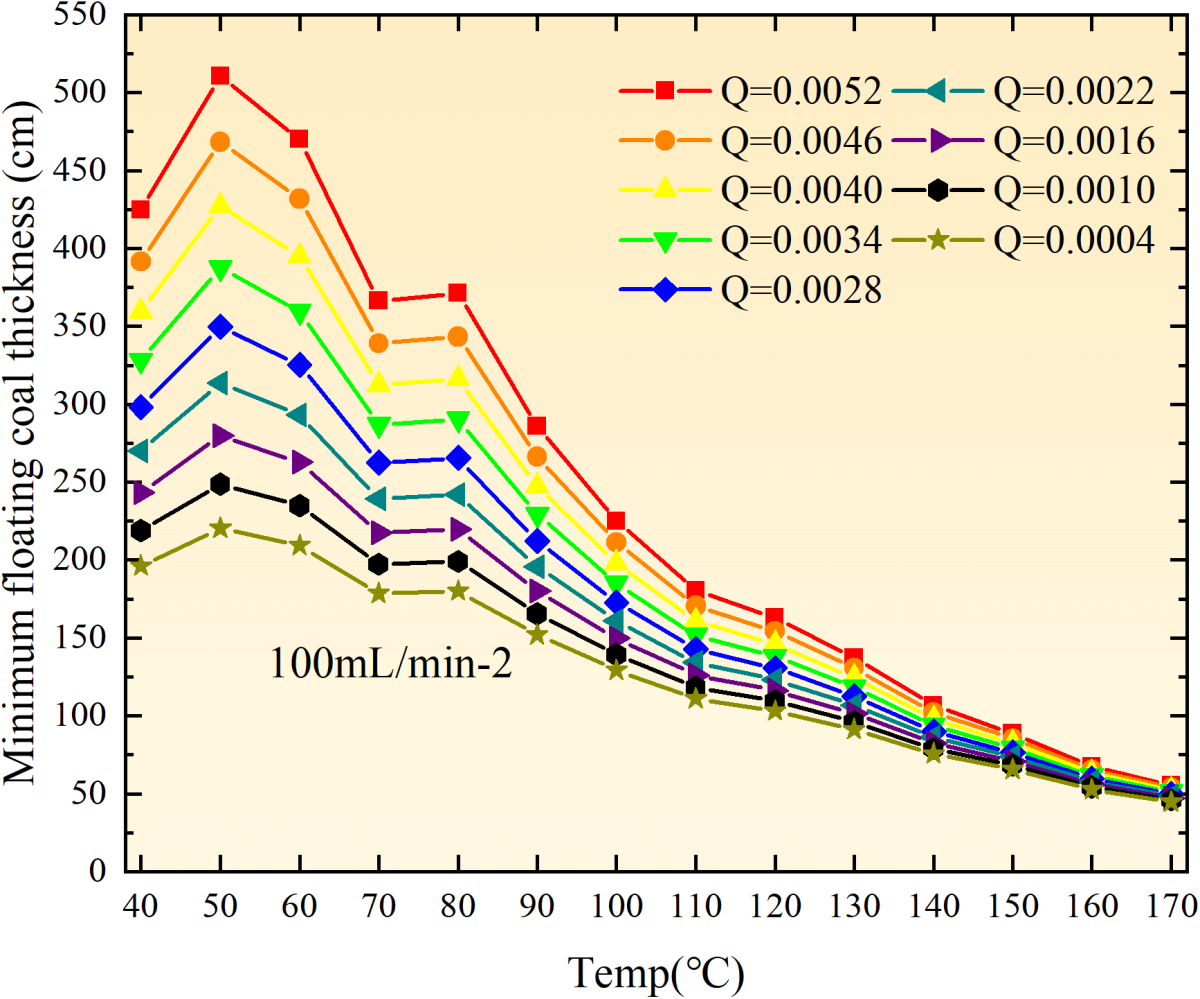
The minimum thickness of float coal under different air leakage intensities.

**Fig 18:**
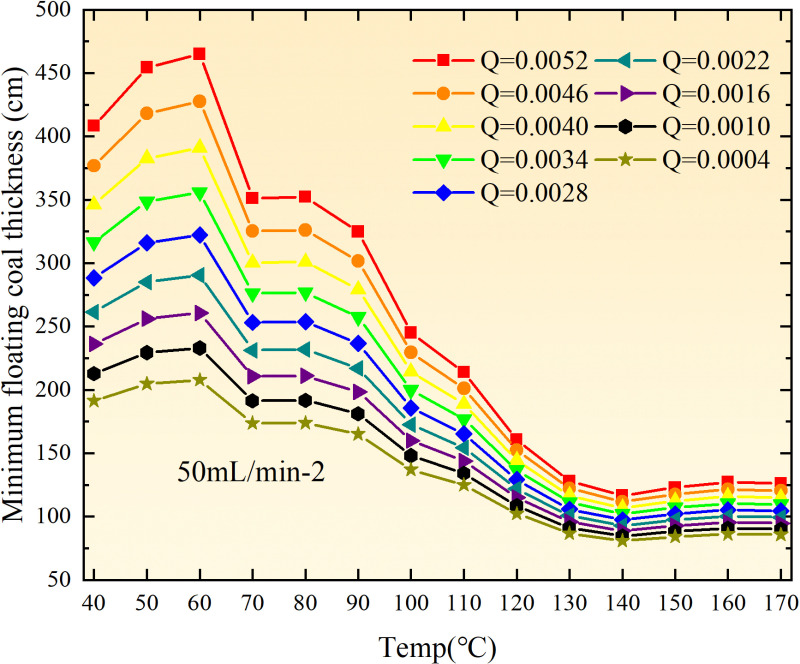
The minimum thickness of float coal under different air leakage intensities.

**Fig 19:**
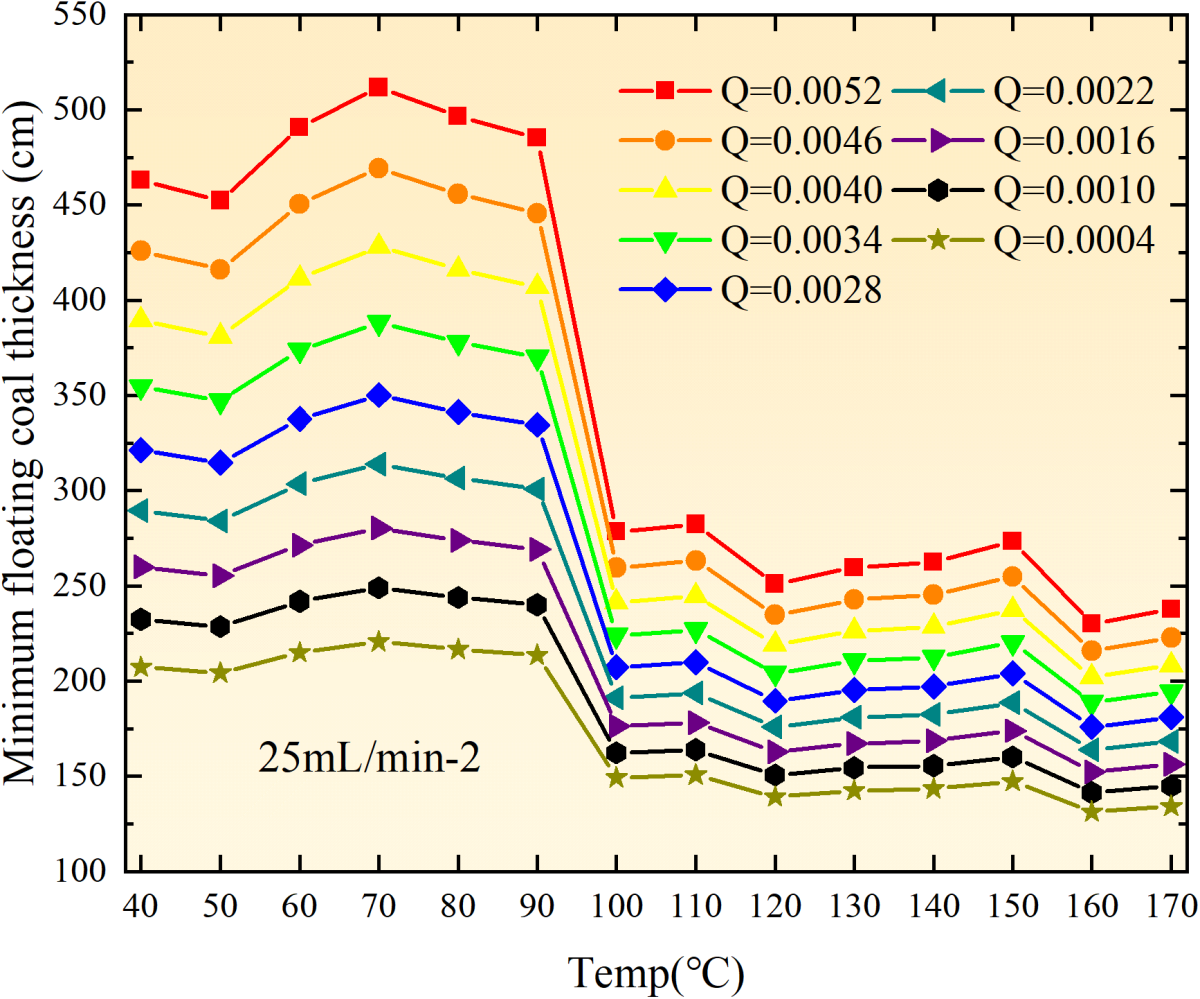
The minimum thickness of float coal under different air leakage intensities.

**Fig 20:**
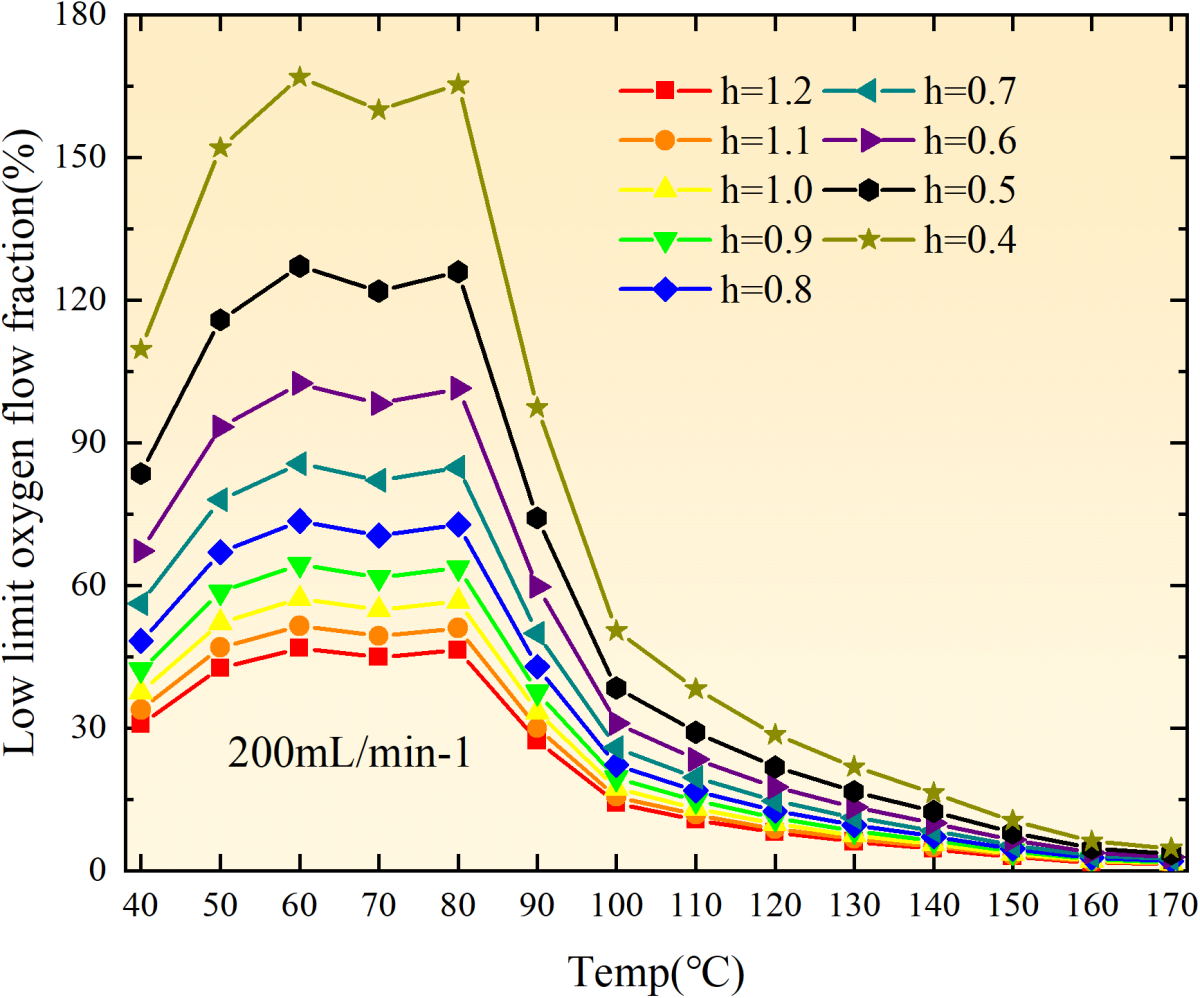
The lower limit of oxygen concentration of coal under different thicknesses of residual coal.

**Fig 21:**
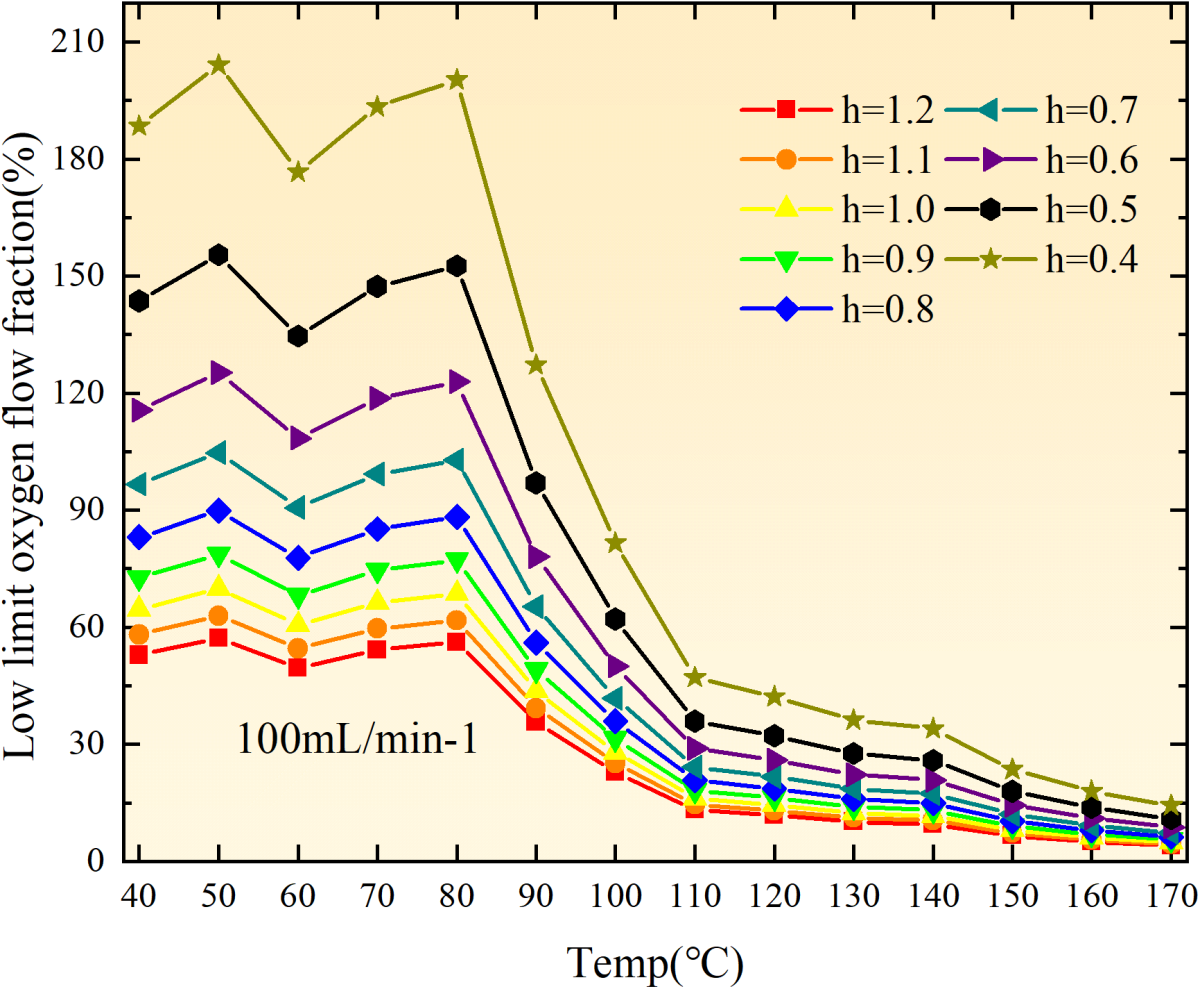
The lower limit of oxygen concentration of coal under different thicknesses of residual coal.

**Fig 22:**
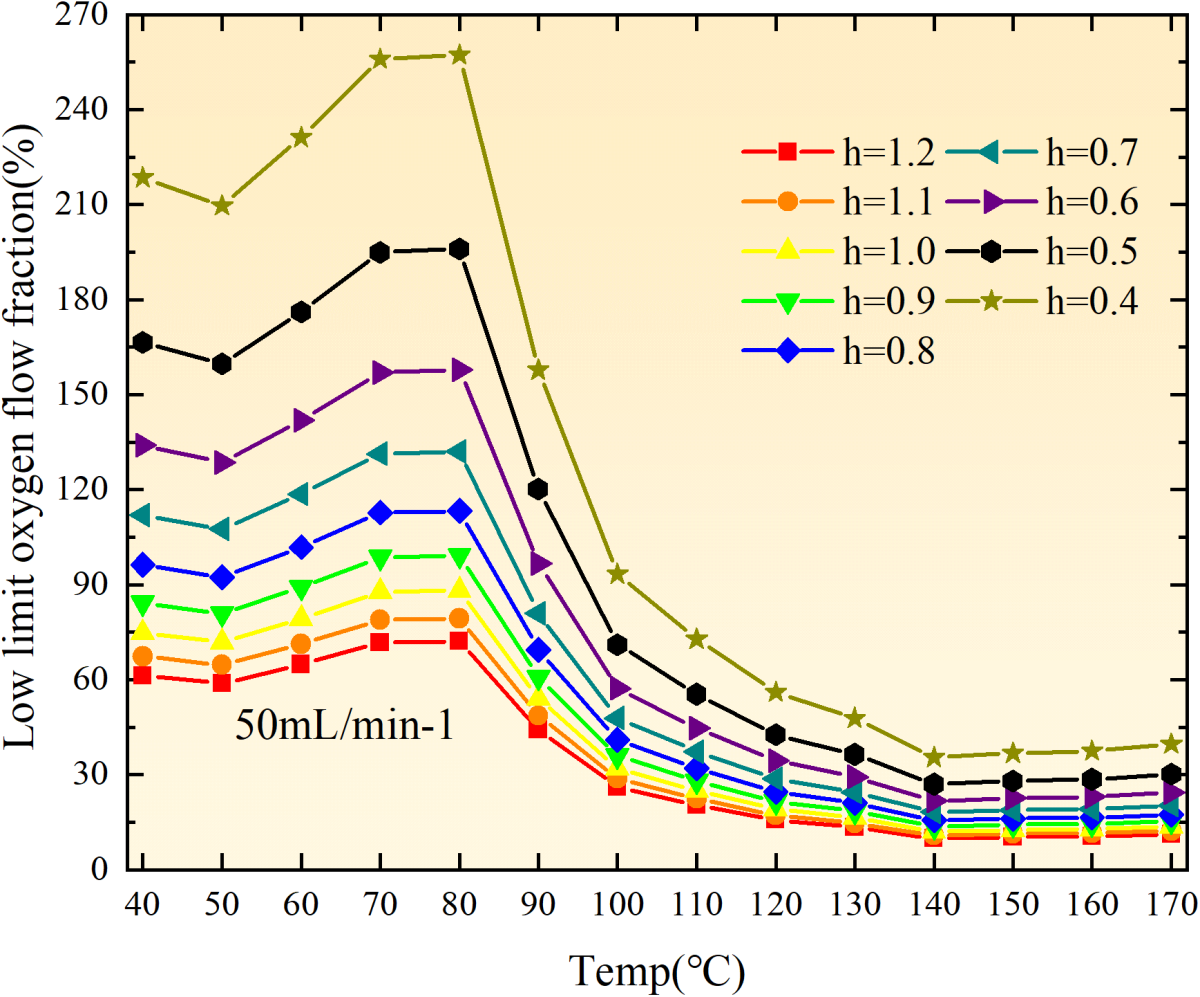
The lower limit of oxygen concentration of coal under different thicknesses of residual coal.

**Fig 23:**
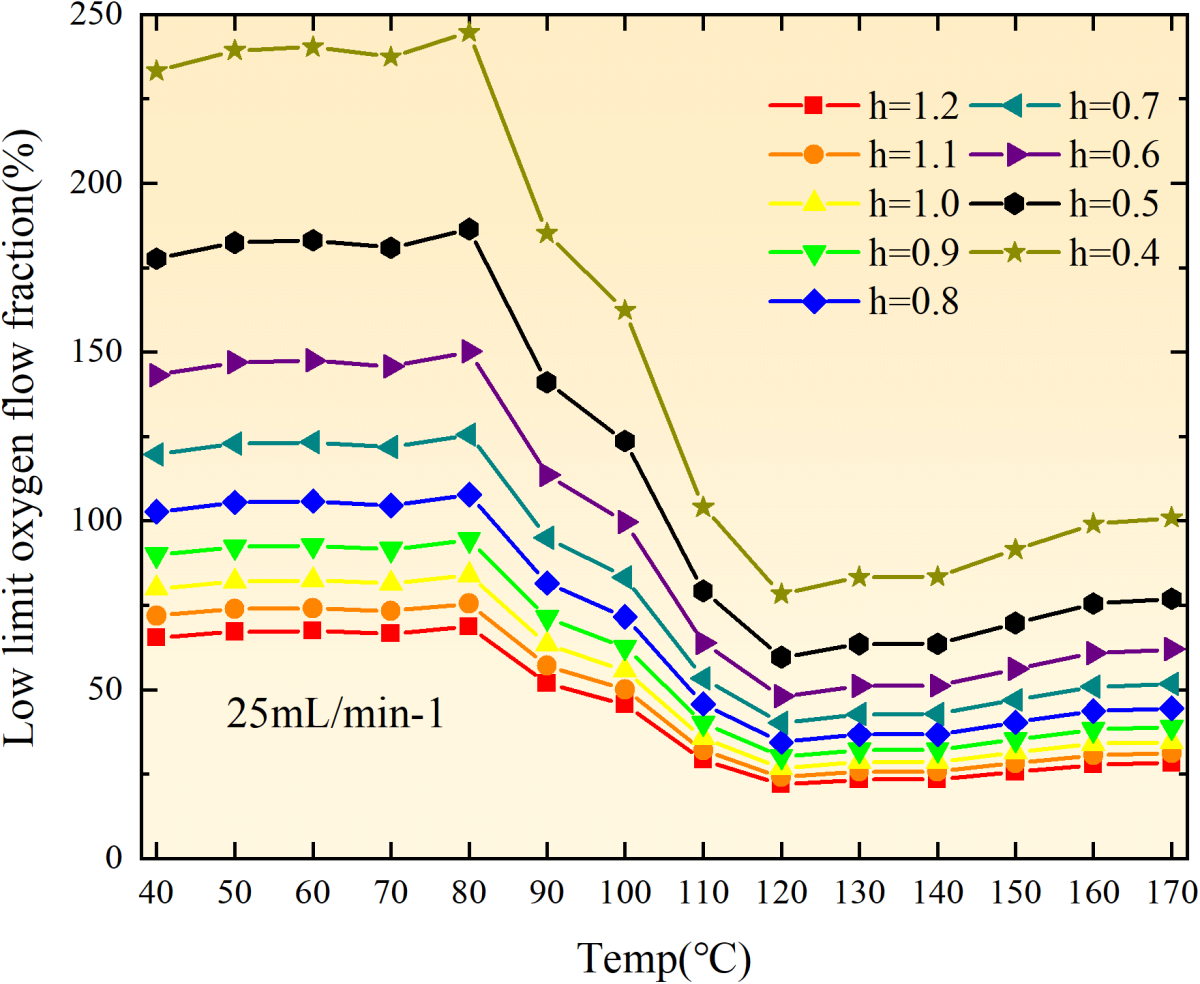
The lower limit of oxygen concentration of coal under different thicknesses of residual coal.

**Fig 24:**
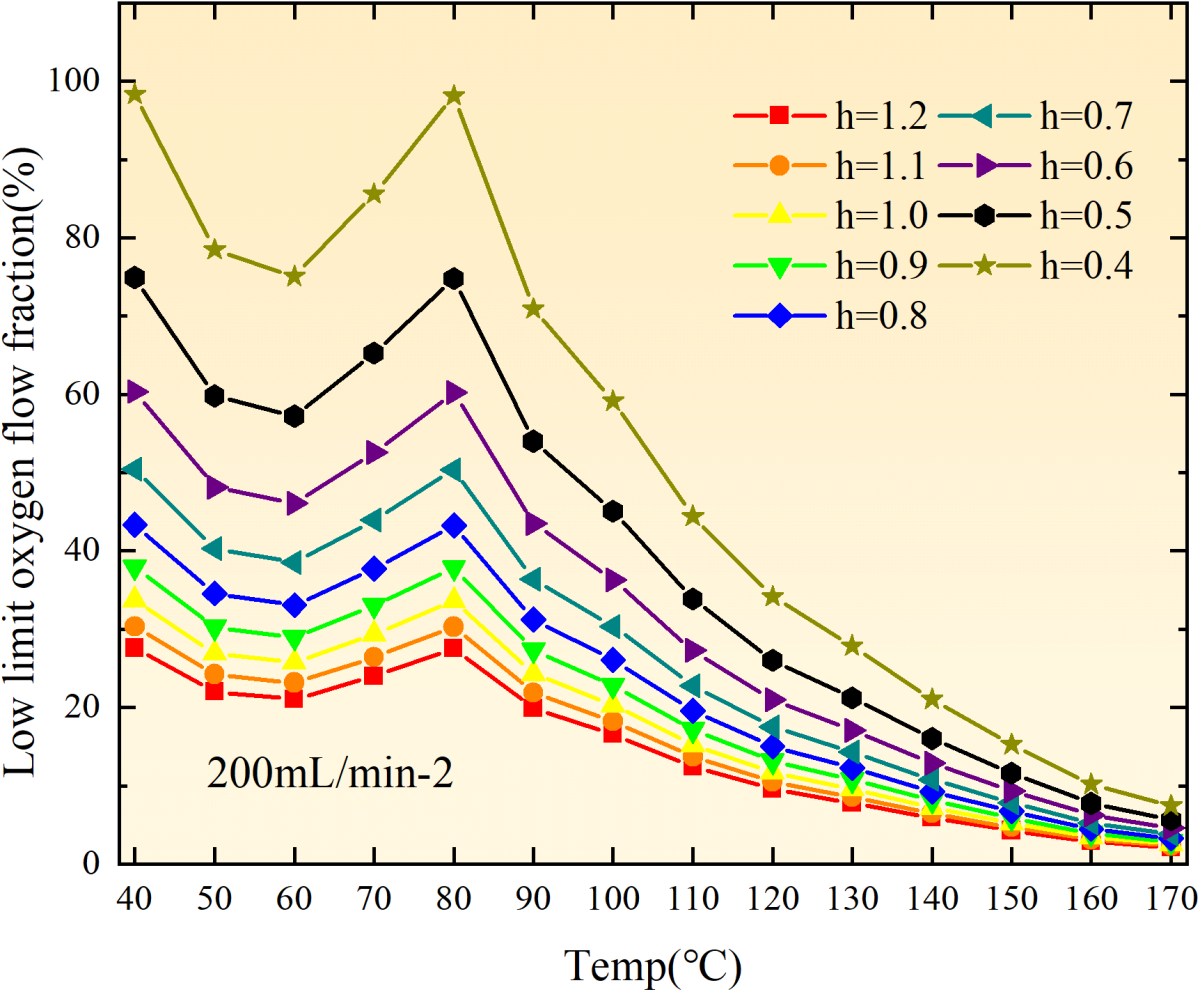
The lower limit of oxygen concentration of coal under different thicknesses of residual coal.

**Fig 25:**
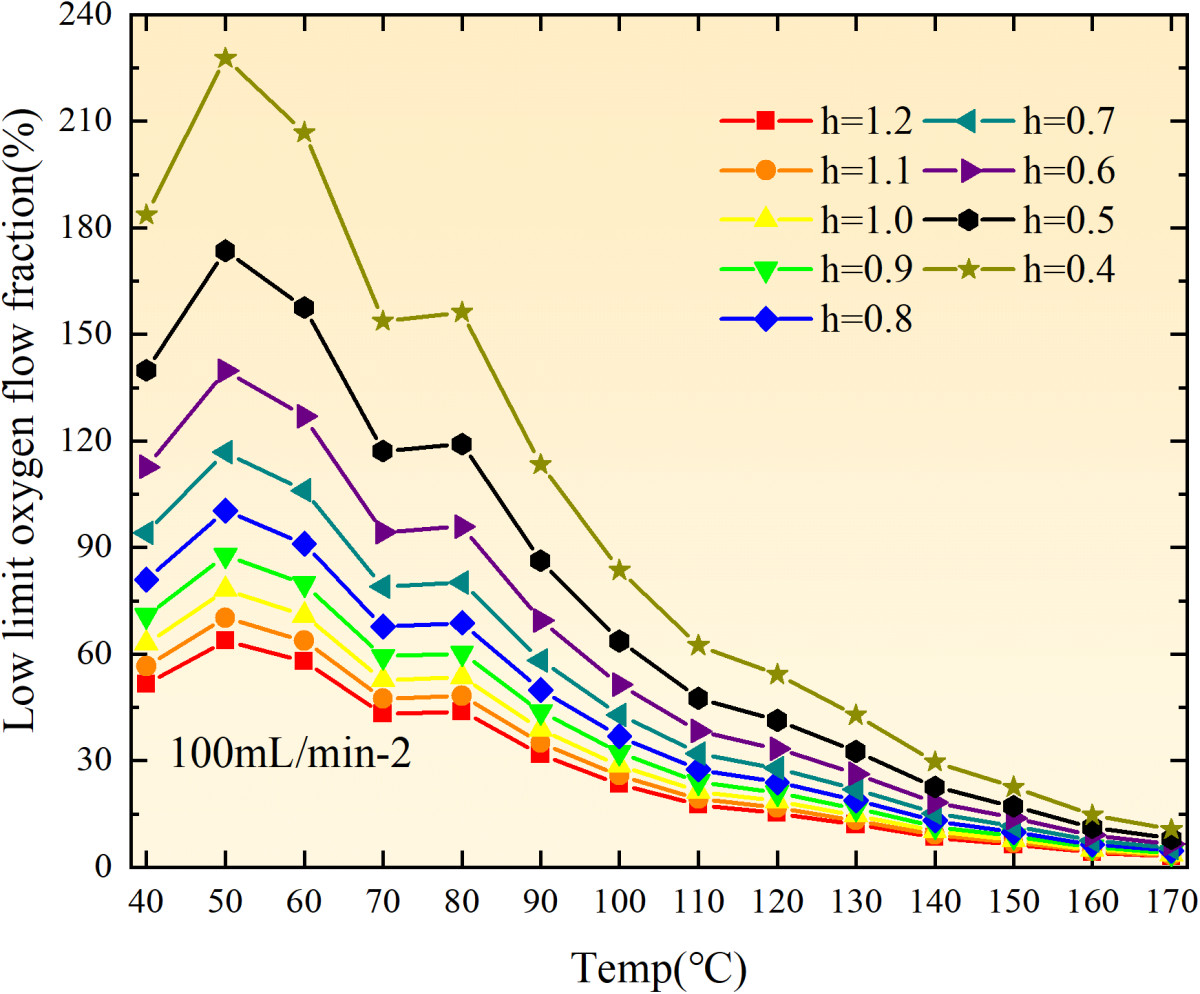
The lower limit of oxygen concentration of coal under different thicknesses of residual coal.

**Fig 26:**
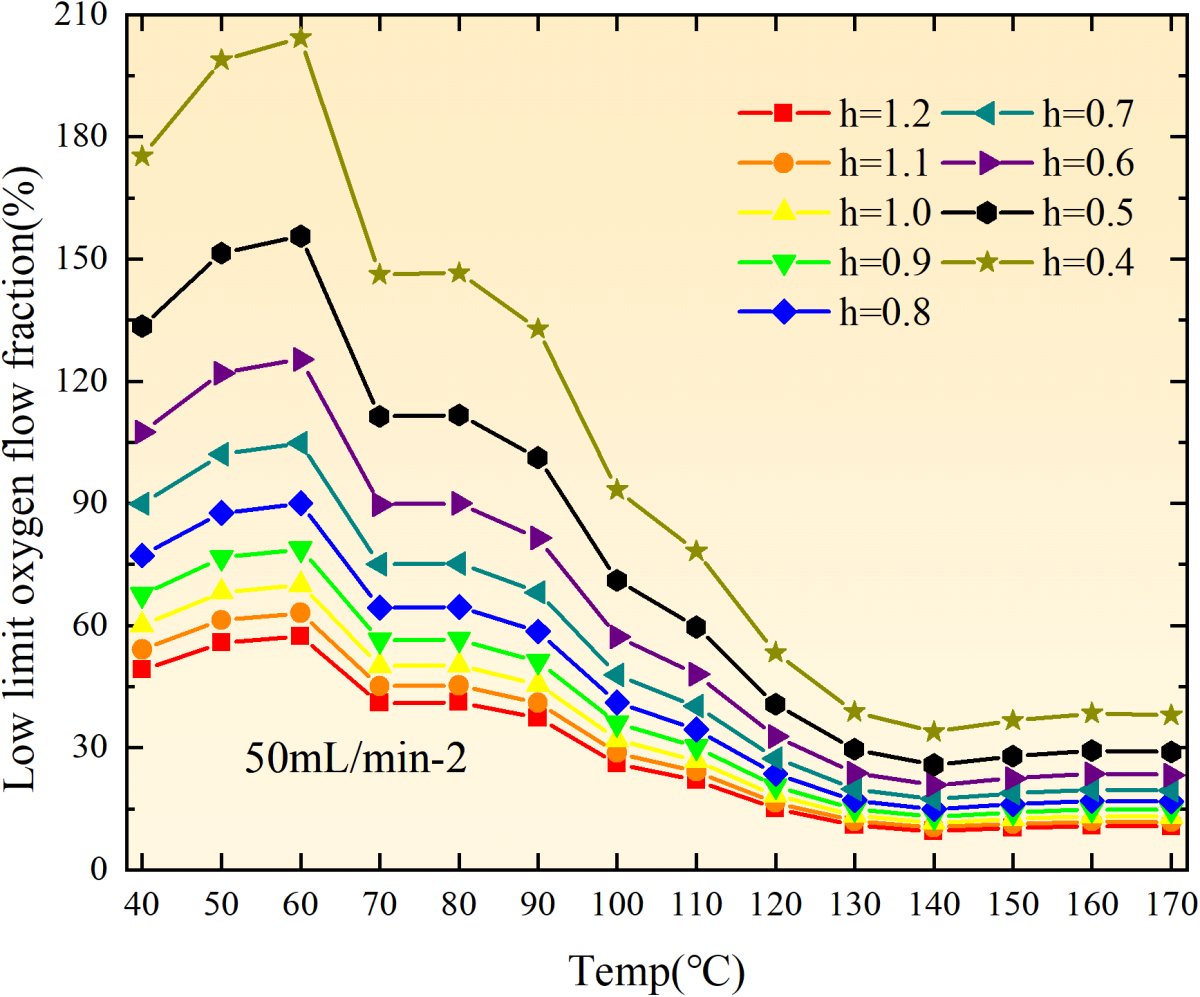
The lower limit of oxygen concentration of coal under different thicknesses of residual coal.

**Fig 27:**
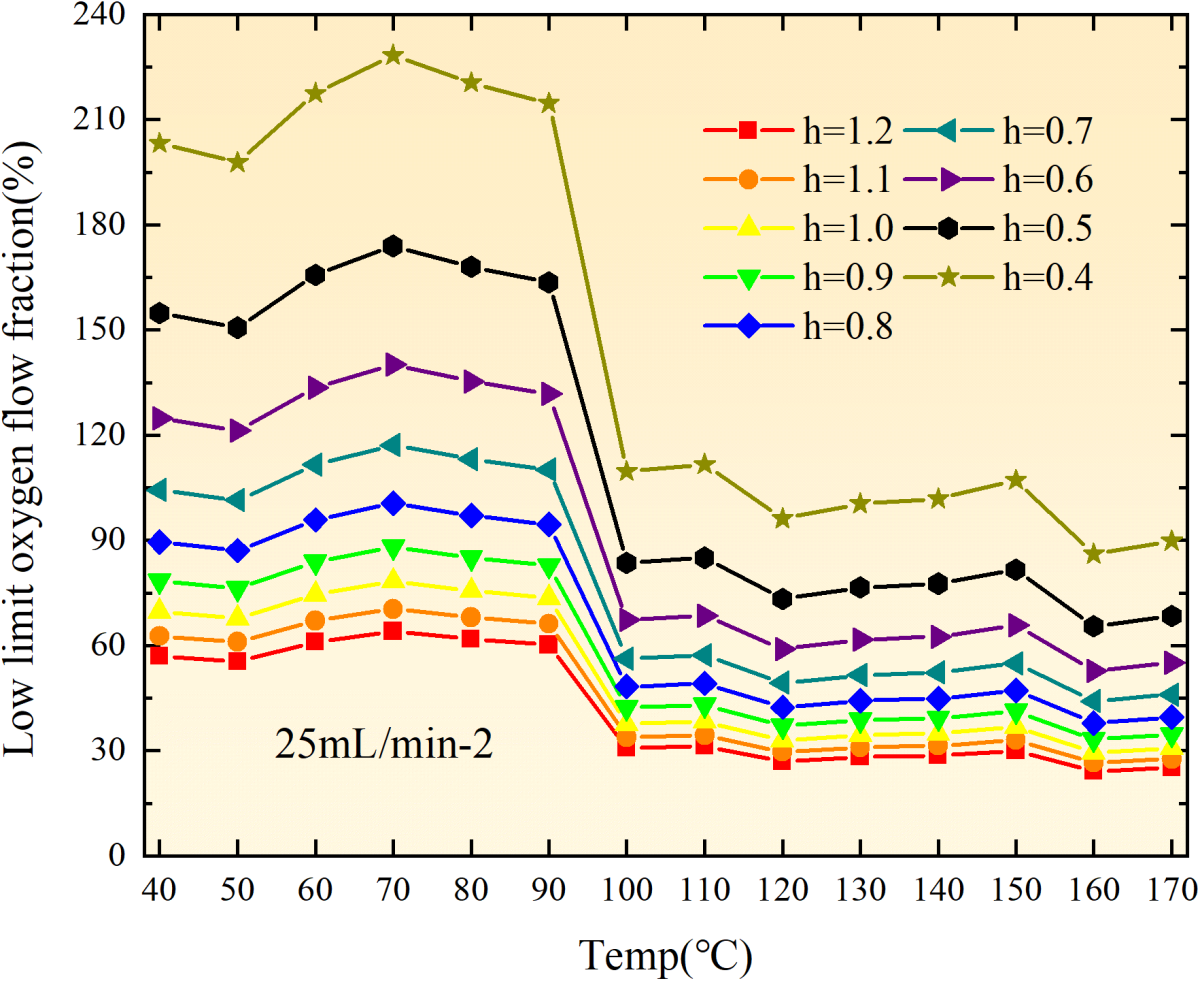
The lower limit of oxygen concentration of coal under different thicknesses of residual coal.

**Fig 28:**
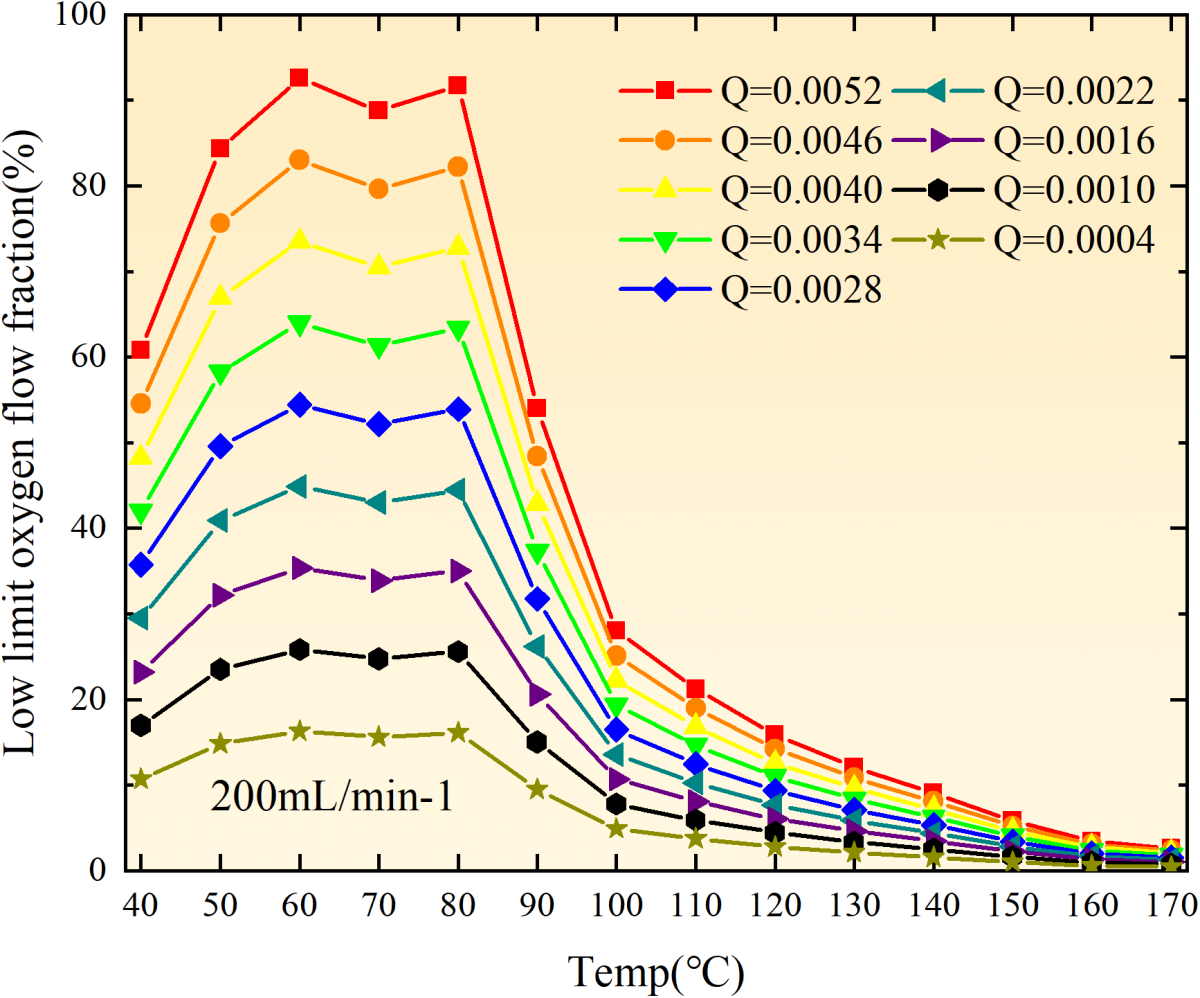
The lower limit of oxygen concentration of coal under different air leakage intensities.

**Fig 29:**
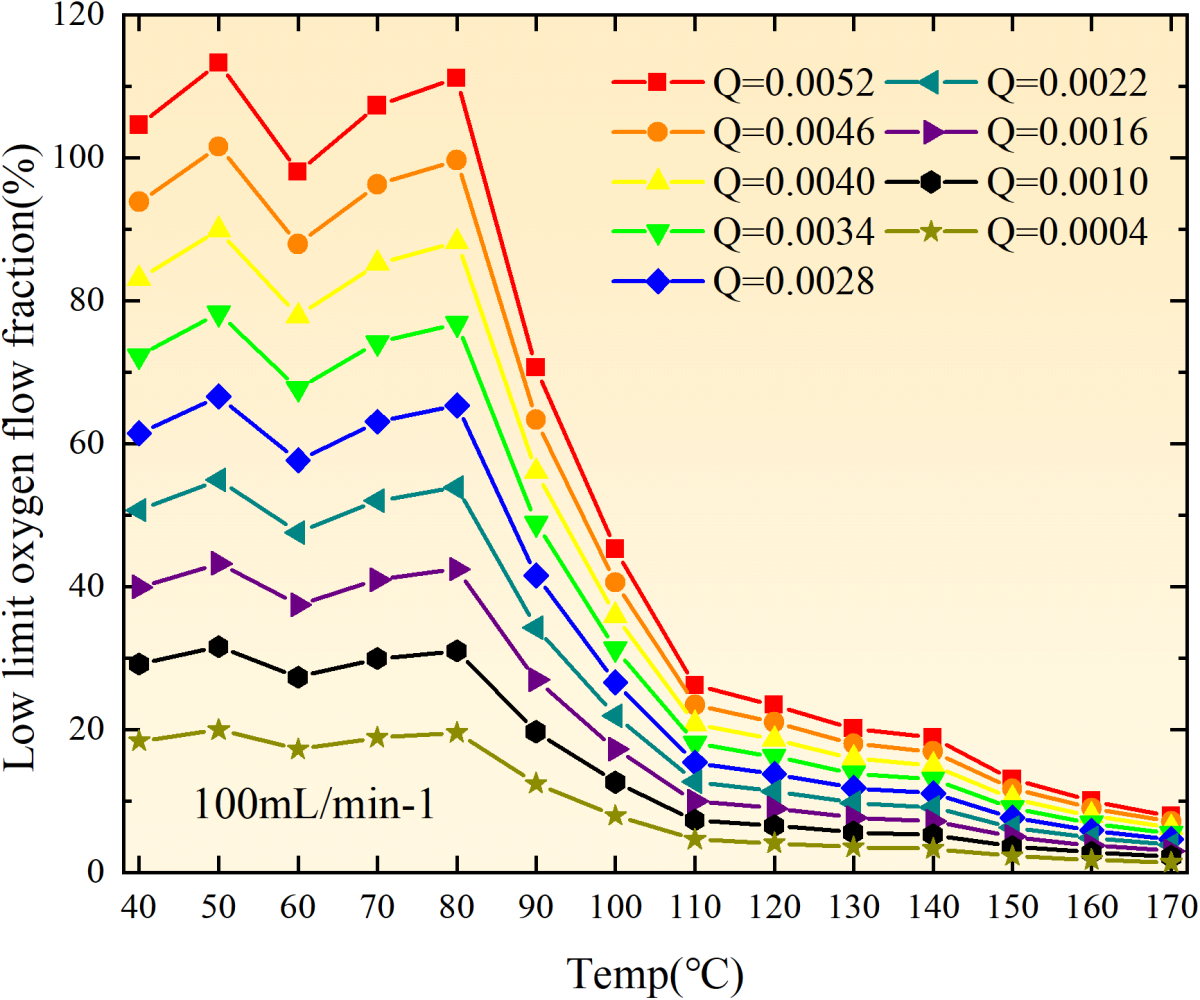
The lower limit of oxygen concentration of coal under different air leakage intensities.

**Fig 30:**
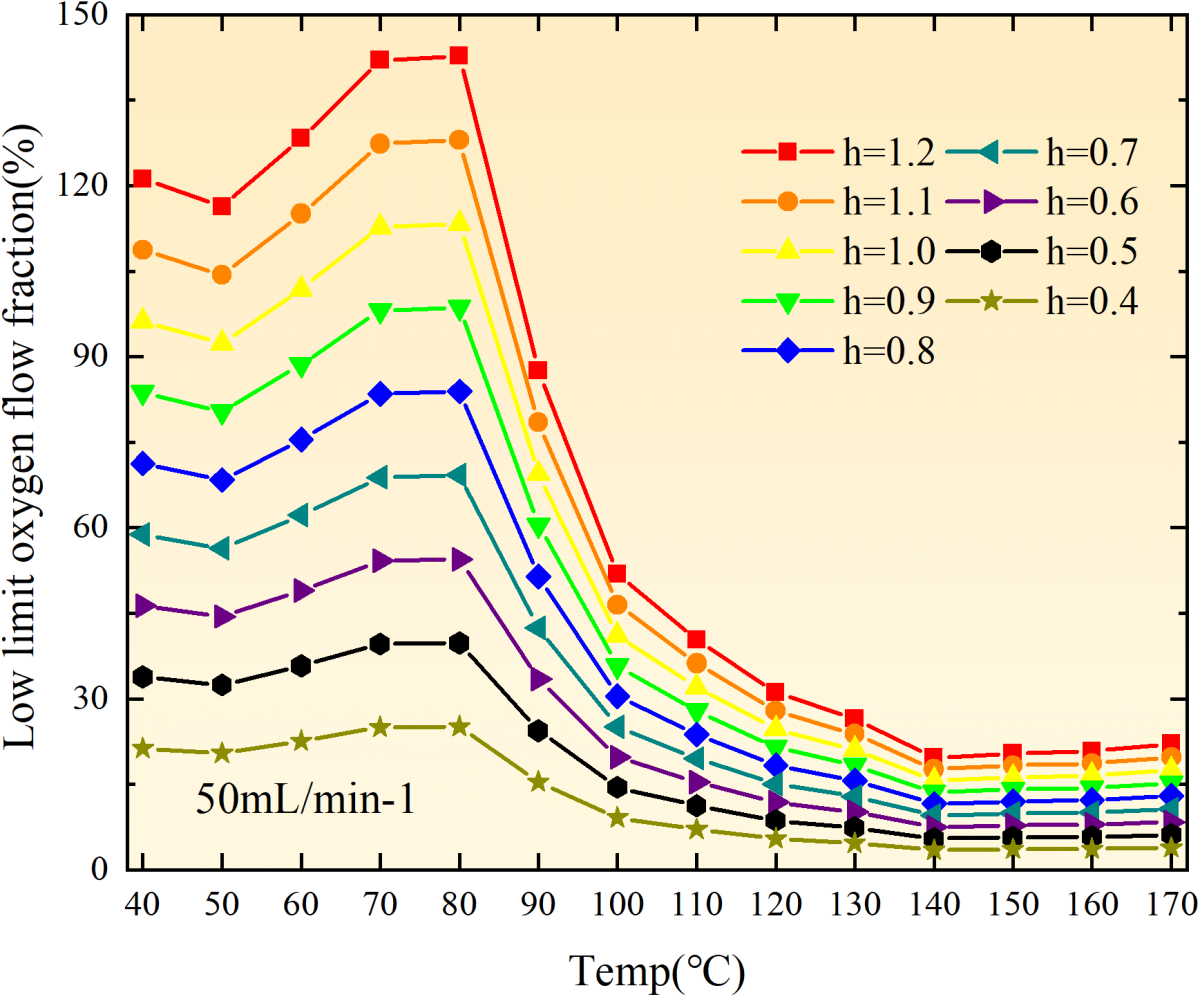
The lower limit of oxygen concentration of coal under different air leakage intensities.

**Fig 31:**
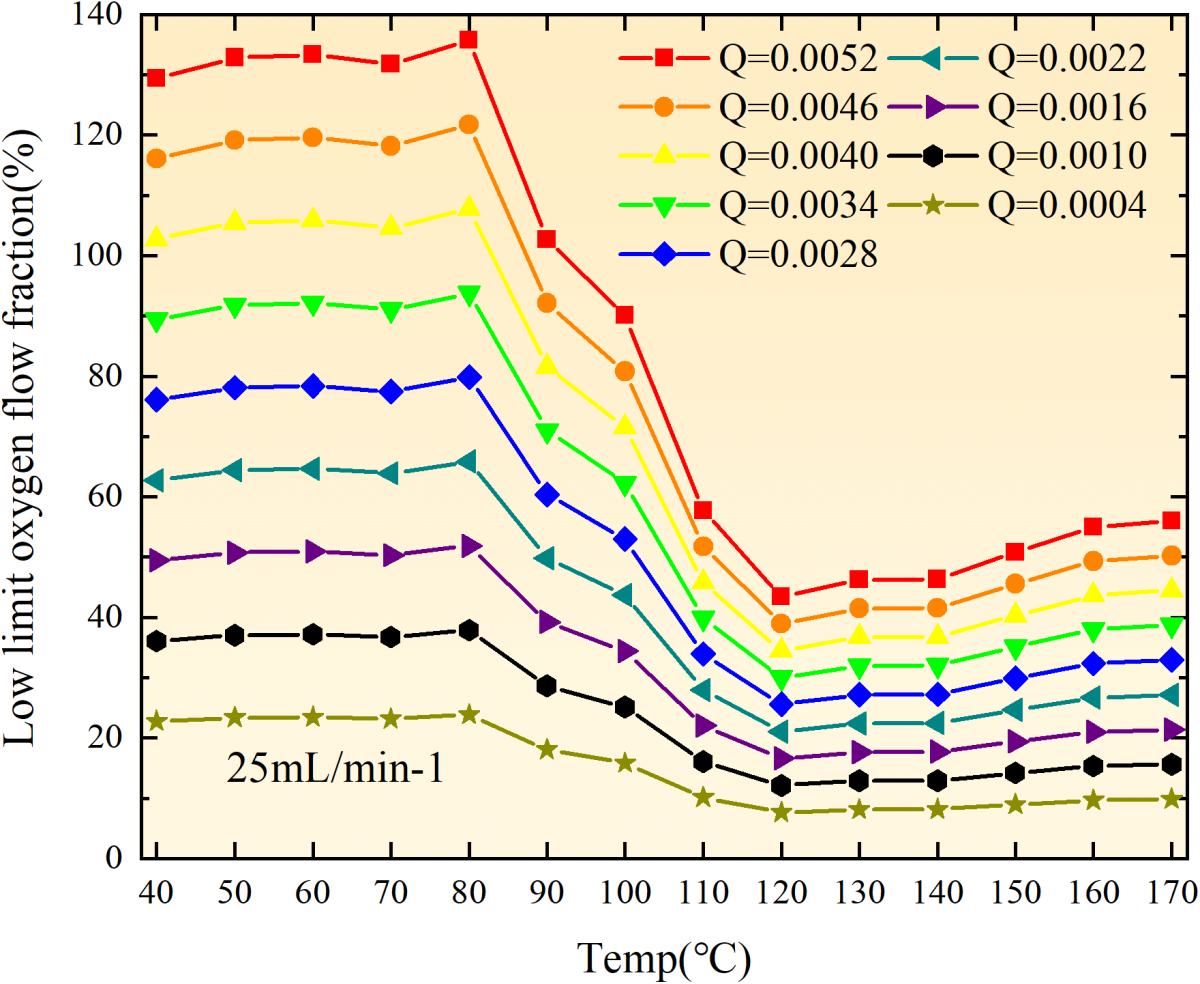
The lower limit of oxygen concentration of coal under different air leakage intensities.

**Fig 32:**
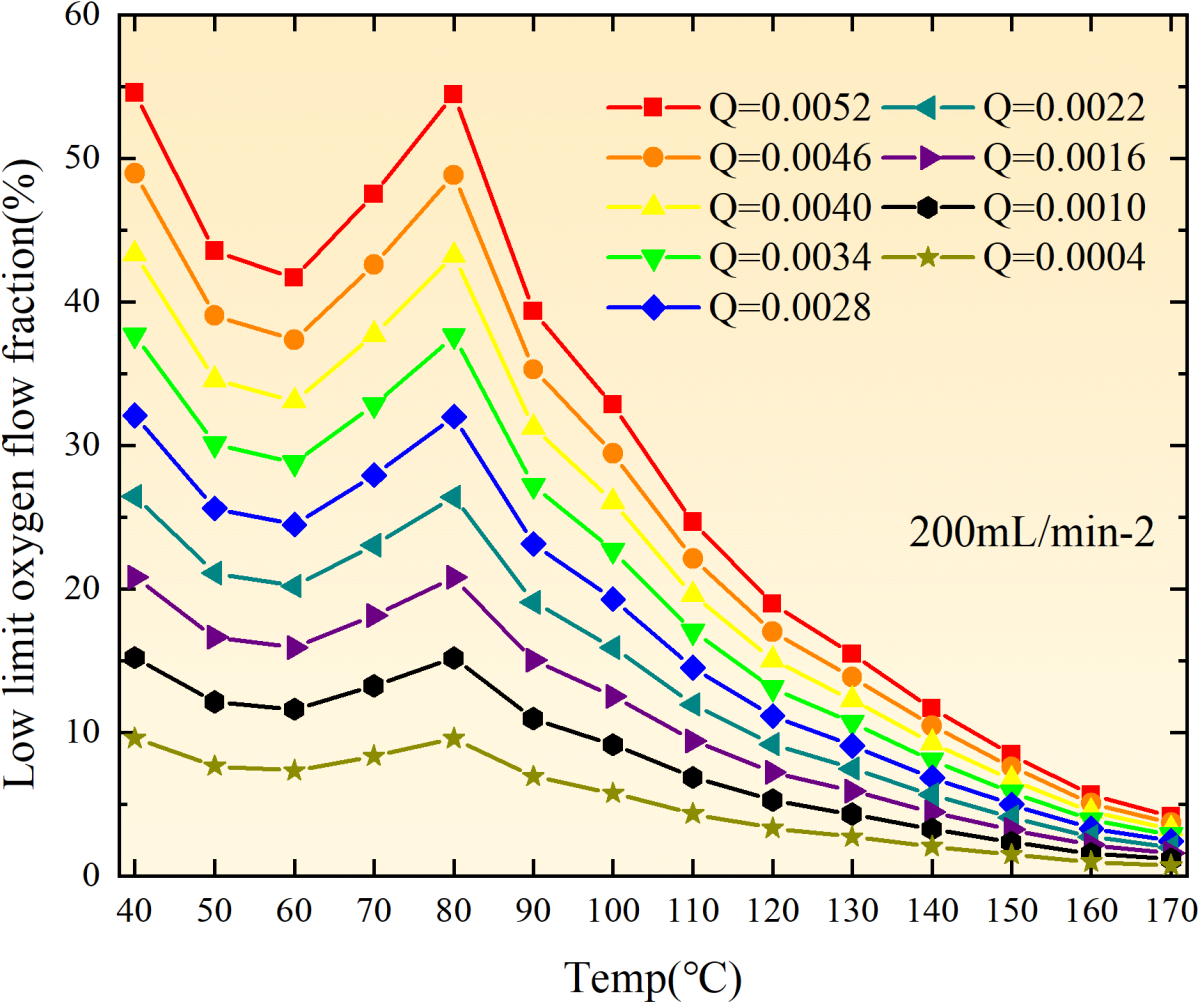
The lower limit of oxygen concentration of coal under different air leakage intensities.

The minimum floating coal thickness increases with the intensity of air leakage, as depicted in Figs 12–43-. Additionally, the minimum floating coal thickness initially increases and then decreases with rising temperature. Moreover, the low limit oxygen flow fraction declines with increasing coal thickness and air leakage intensity but exhibits a trend of first increasing and then decreasing with temperature. The minimum floating coal thickness and the low limit oxygen flow fraction during the oxidation process decrease as the air flow increases, while the low limit oxygen flow fraction increases with increasing air flow at the starting temperature of coal spontaneous combustion (coal temperature 40 °C). During the primary and secondary oxidation process, a higher degree of air leakage results in a reduced amount of residual coal required to trigger spontaneous combustion. This leads to lower oxygen demand and an increased range of air leakage intensity that can initiate spontaneous combustion. Consequently, there is a larger area at risk for spontaneous combustion in goaf, resulting in higher risks.

**Fig 33:**
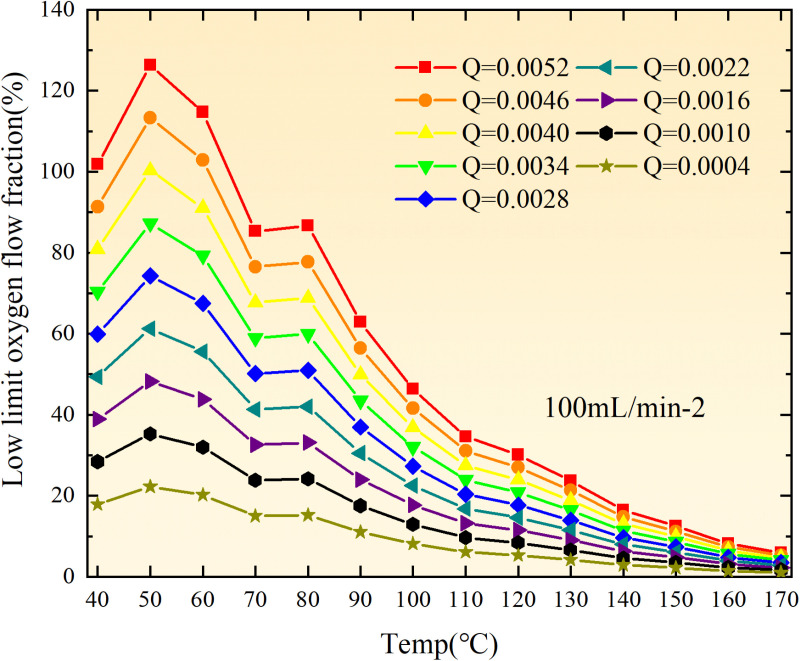
The lower limit of oxygen concentration of coal under different air leakage intensities.

**Fig 34:**
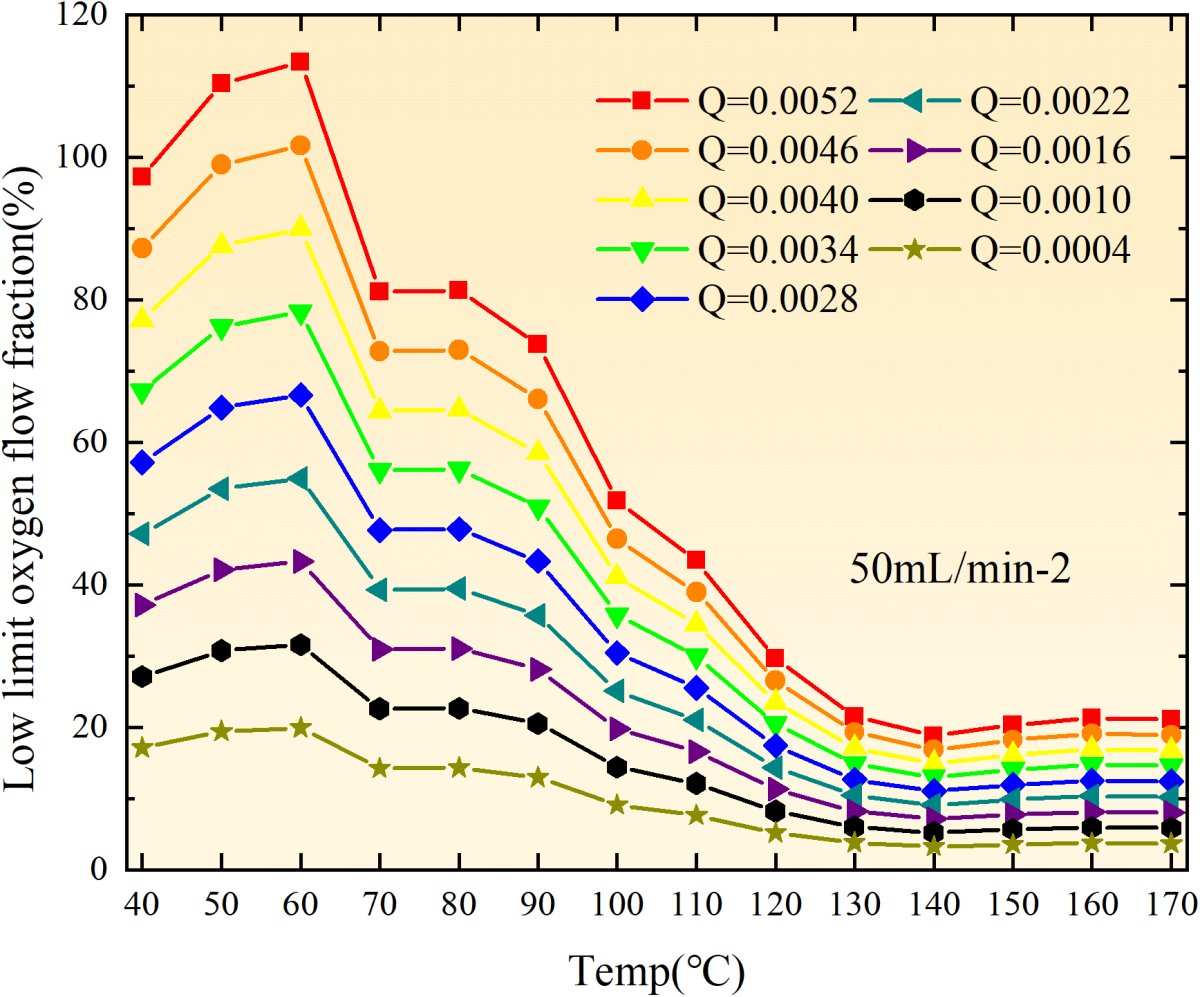
The lower limit of oxygen concentration of coal under different air leakage intensities.

**Fig 35:**
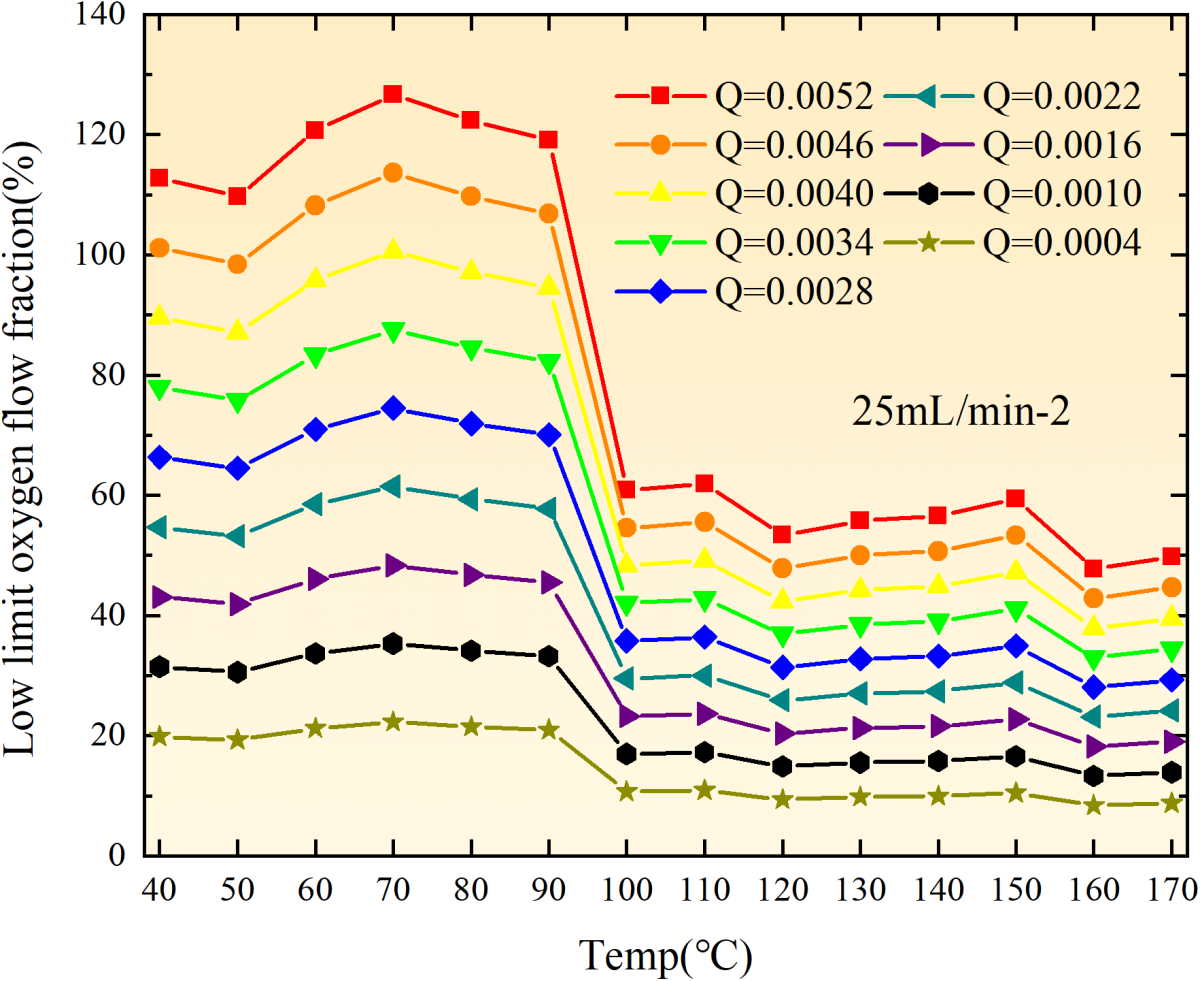
The lower limit of oxygen concentration of coal under different air leakage intensities.

**Fig 36:**
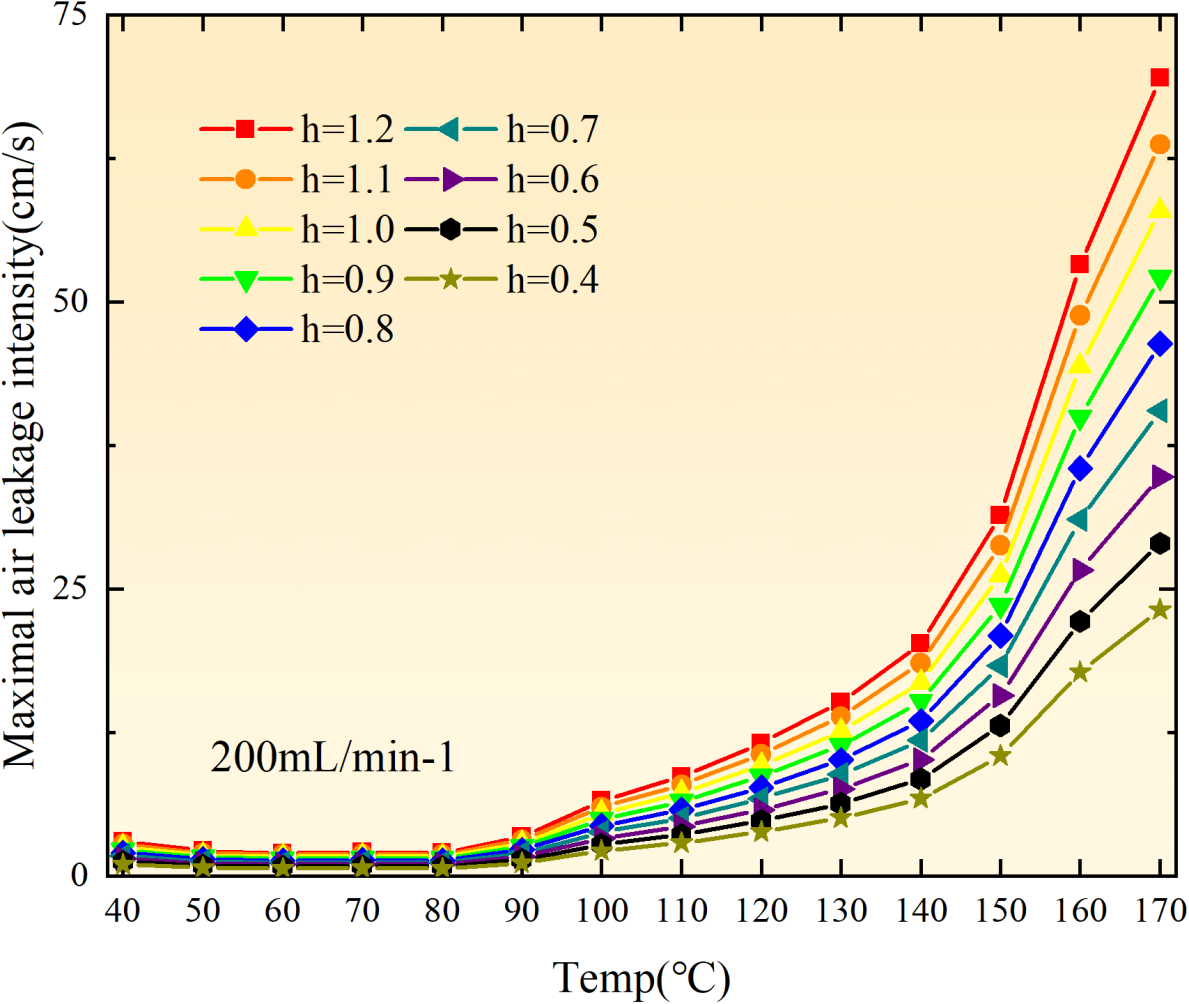
Maximal air leakage intensity under different thickness of the loose coal body.

**Fig 37:**
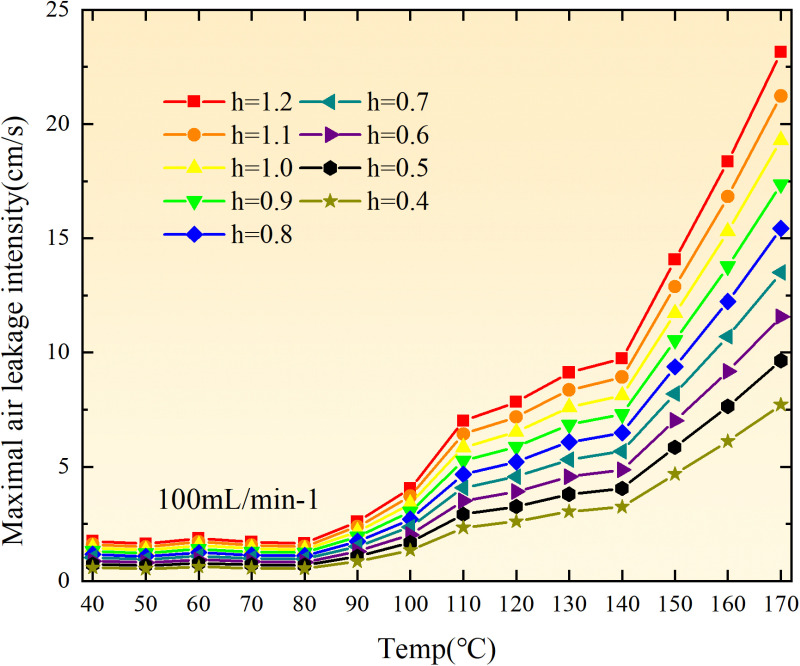
Maximal air leakage intensity under different thickness of the loose coal body.

**Fig 38:**
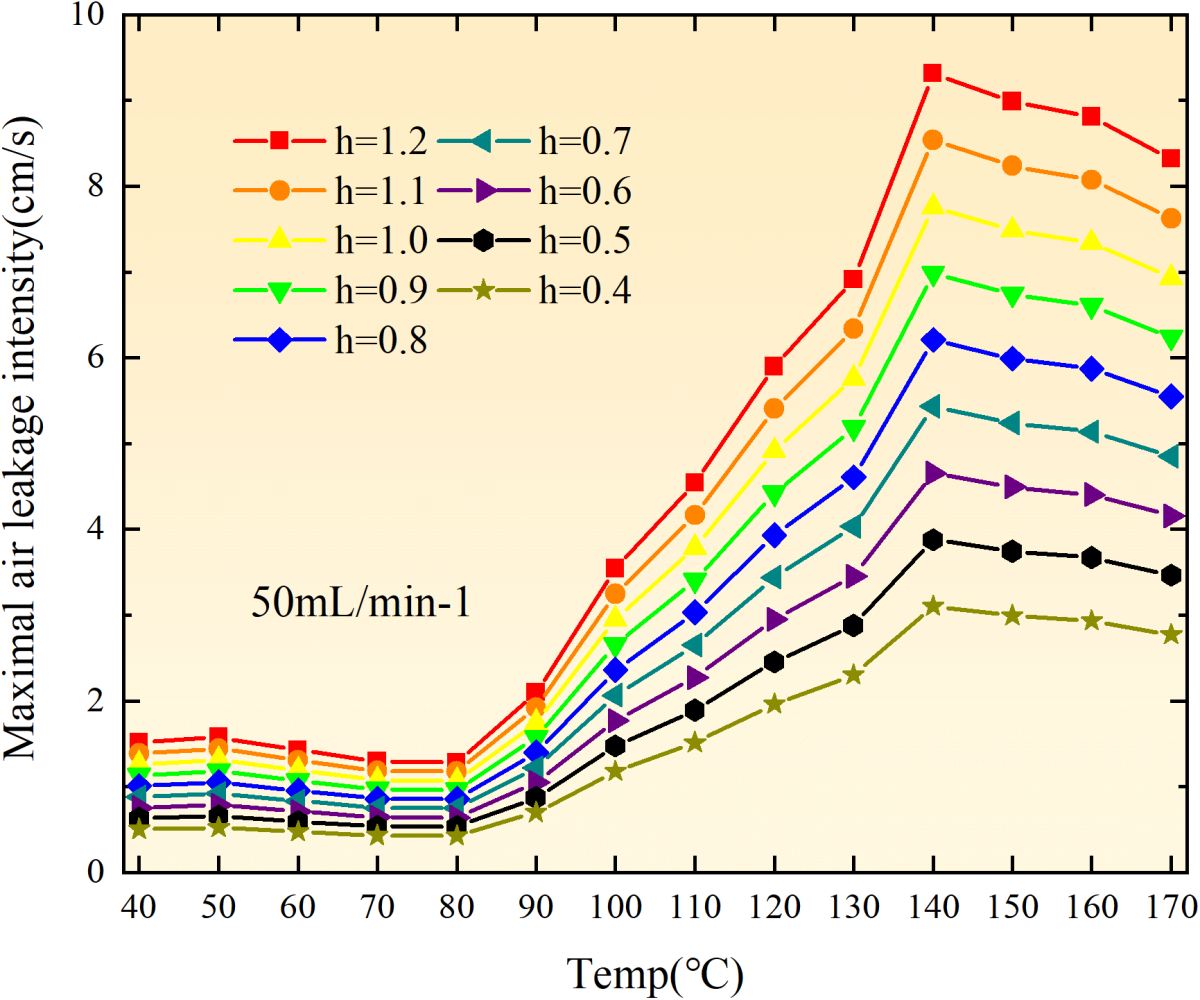
Maximal air leakage intensity under different thickness of the loose coal body.

**Fig 39:**
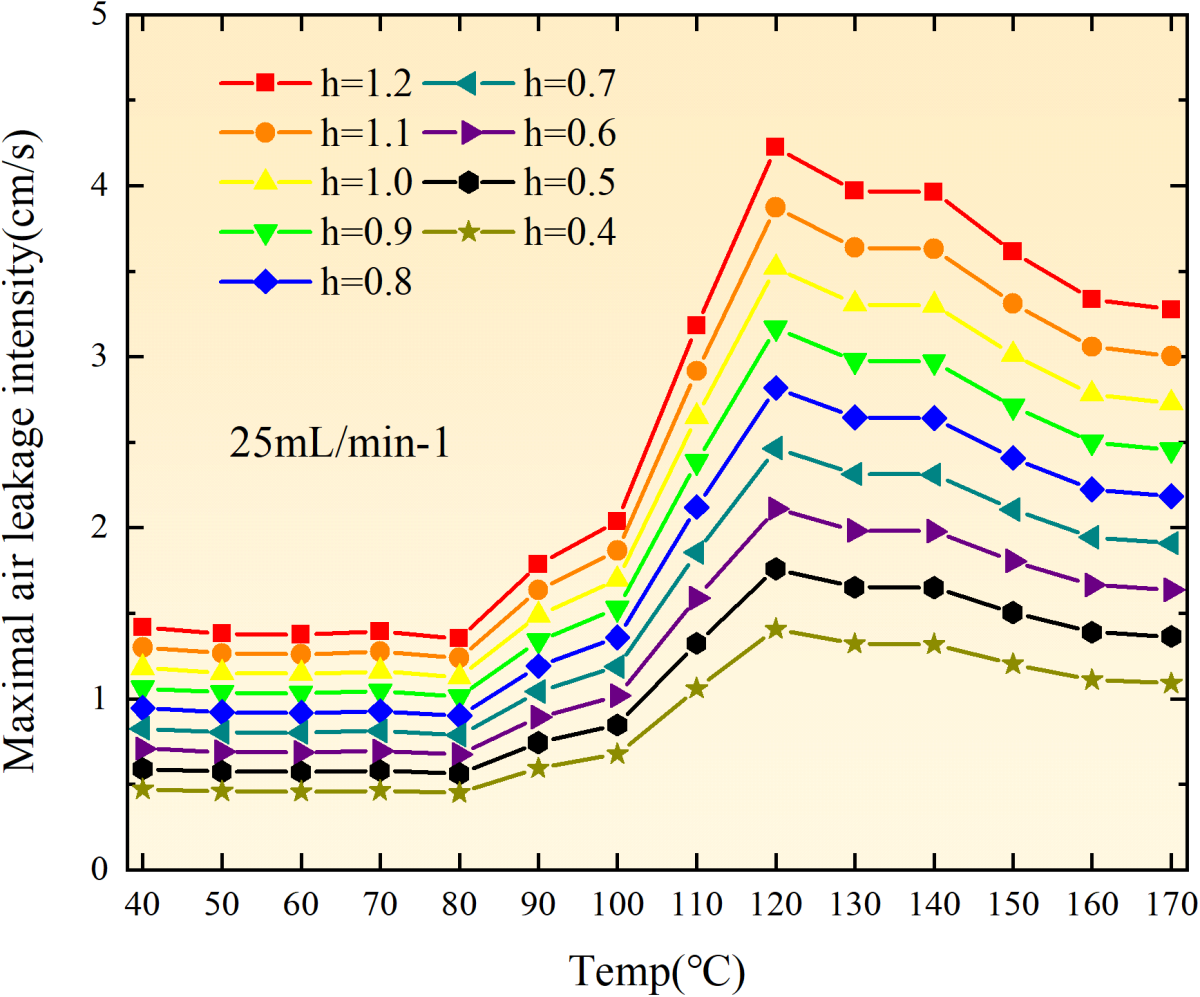
Maximal air leakage intensity under different thickness of the loose coal body.

**Fig 40:**
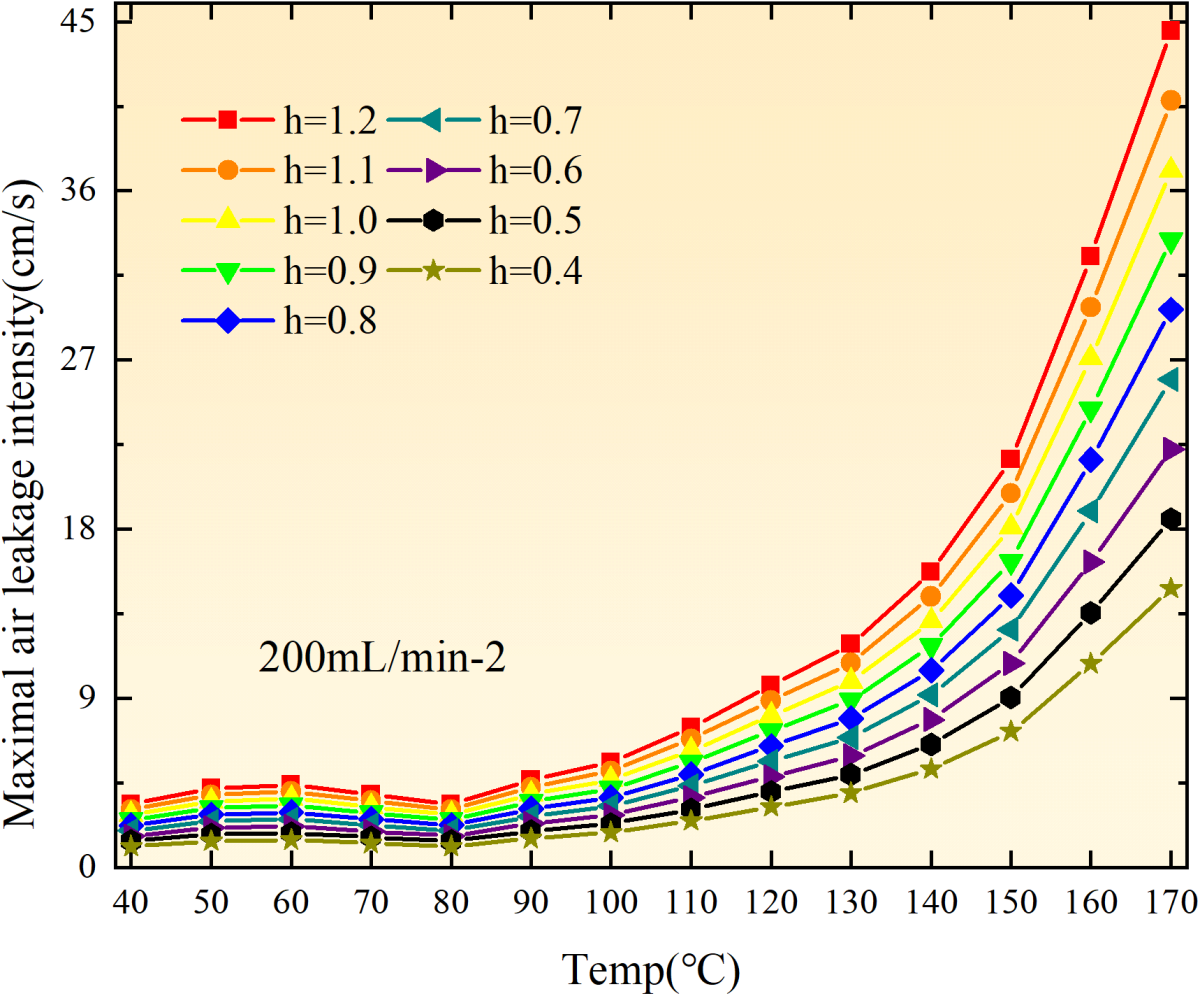
Maximal air leakage intensity under different thickness of the loose coal body.

**Fig 41:**
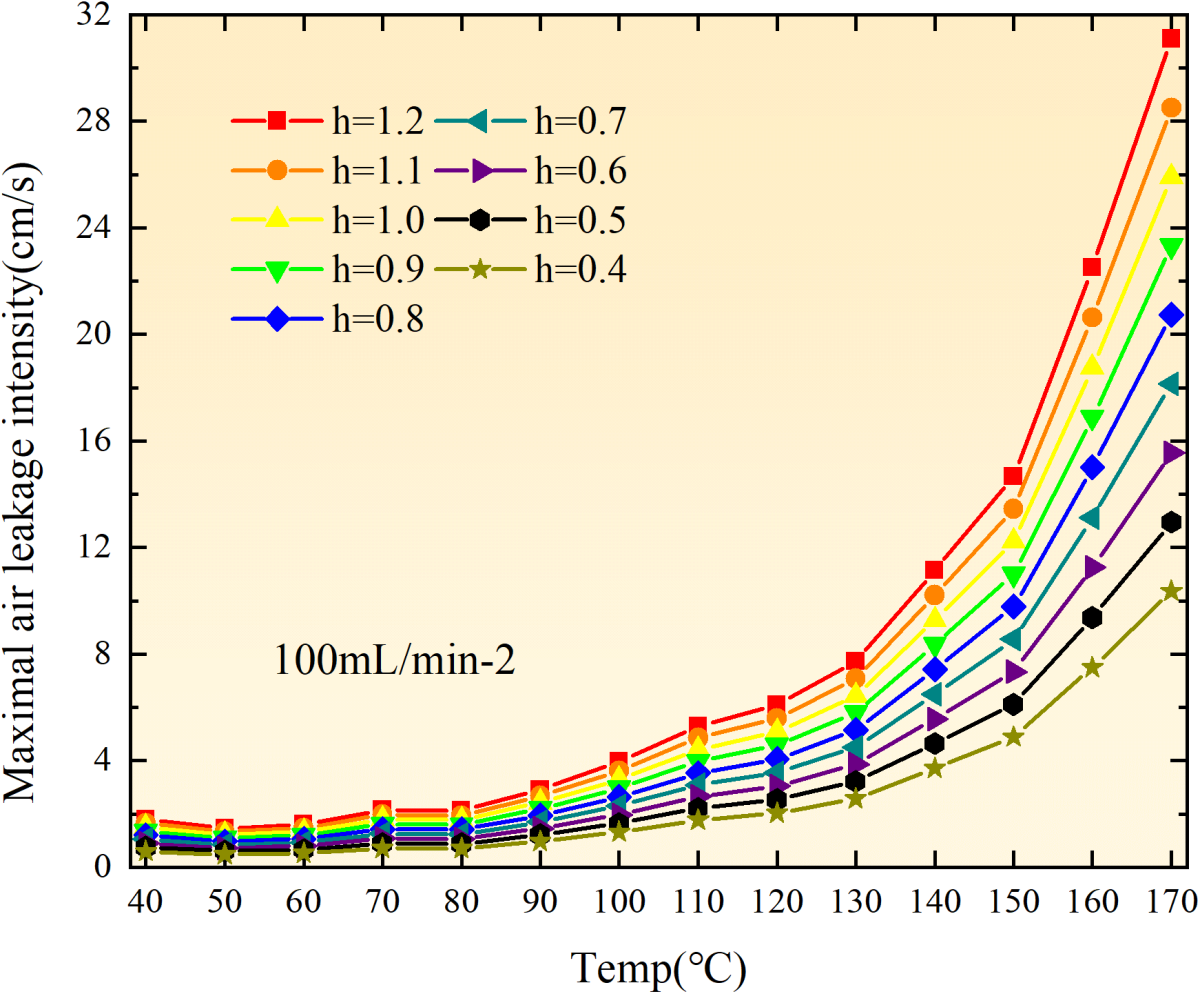
Maximal air leakage intensity under different thickness of the loose coal body.

**Fig 42:**
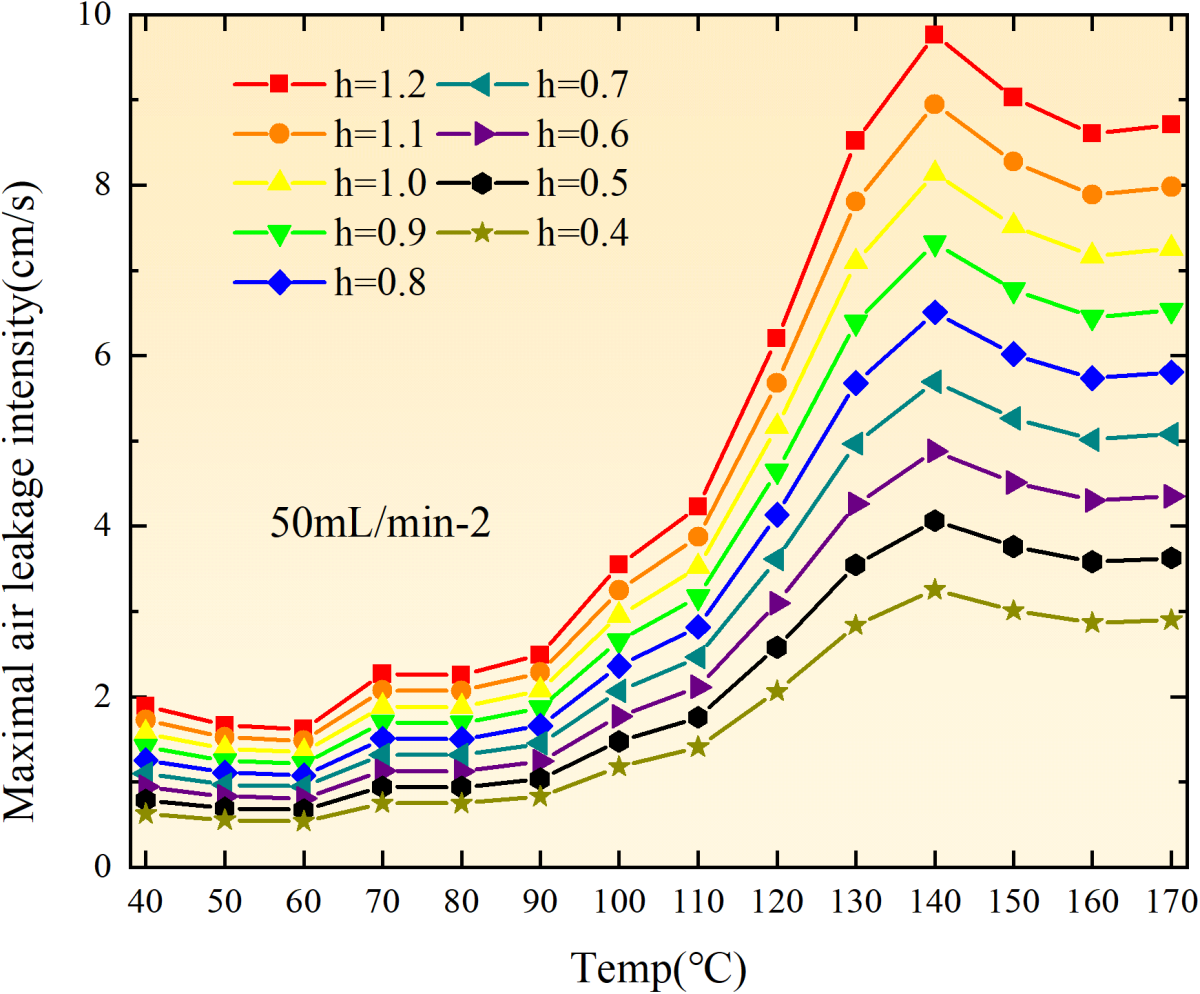
Maximal air leakage intensity under different thickness of the loose coal body.

**Fig 43:**
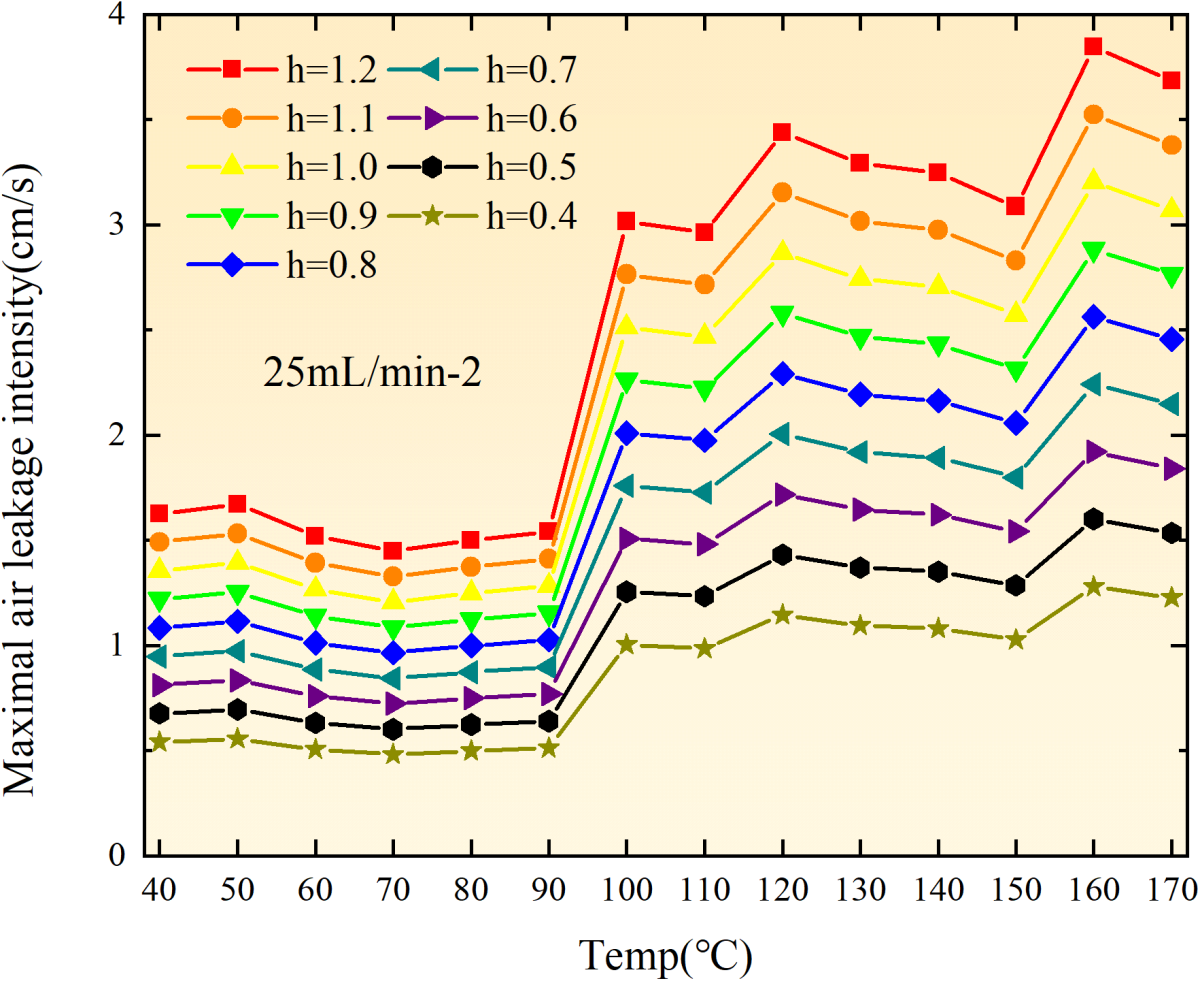
Maximal air leakage intensity under different thickness of the loose coal body.

Compared to primary and secondary oxidation, the coal samples subjected to the same experimental air flow exhibit lower minimum floating coal thickness and low limit oxygen flow fraction during secondary oxidation, while displaying higher maximal air leakage intensity compared to primary oxidation at the onset temperature of spontaneous combustion (coal temperature 40 °C). With an increase in oxidation times, the amount of residual coal required to trigger spontaneous combustion decreases, resulting in a lower oxygen demand. This leads to a wider range of air leakage intensities that can initiate spontaneous combustion and expands the dangerous areas where goaf spontaneous combustion can occur, consequently increasing the risk of such incidents.

The sustainability of coal spontaneous combustion phenomenon also depends on the minimum floating coal thickness, the low limit oxygen flow fraction, and the maximal air leakage intensity. Only when both the minimum floating coal thickness and low limit oxygen flow fraction exceed their respective maximum values, while the maximal air leakage intensity remains below its minimum value, can this natural occurrence be sustained. The primary oxidation process, as illustrated in Figs 12–35, reveals that under identical conditions, the coal sample with an experimental air flow of 50mL/min exhibits the largest minimum floating coal thickness and low limit oxygen flow fraction. It is followed by the coal sample with an air flow of 25 mL/min and then by the one with 100mL/min. Conversely, the coal sample exposed to an air flow of 200 mL/min demonstrates the smallest maximum value. These findings indicate that sustaining spontaneous combustion during primary oxidation is most challenging for the coal sample subjected to an air flow of 50 mL/min, followed by those with flows of 25 mL/min and 100 mL/min respectively. On the other hand, sustaining spontaneous combustion becomes easier for samples exposed to an air flow of 200 mL/min. During secondary oxidation under identical conditions, it is observed that among all tested samples, the minimum floating coal thickness and low limit oxygen flow fraction are highest for a coal sample subjected to an experimental air flow of 100 mL/min. This is followed by samples exposed to flows of both at a rate of only half (i.e.,50 mL/min) and subsequently at a rate of only one-fourth (i.e.,25 mL/min). Finally, the lowest values are found in relation to a sample treated with a maximum airflow rate (i.e.,200 mL/min). These results suggest that as airflow increases during secondary oxidation processes: less residual coal is required for continued self-combustion; lower oxygen concentrations are necessary; wider natural ranges can be sustained; ultimately leading to stronger continuity in spontaneous combustion.

By comparing the maximum values of the minimum floating coal thickness in the primary and secondary oxidation processes, it is observed that, except for the coal samples with an air flow of 100mL/min, all maximum values of coal samples in the secondary oxidation process are smaller than those in the primary oxidation process. Furthermore, both primary and secondary oxidation processes exhibit similar maximum values to that of the 100mL/min coal samples. These findings indicate sustained spontaneous combustion of coal during secondary oxidation, requiring a smaller amount of residual coal and lower oxygen concentration. Moreover, this phenomenon leads to a wider range and stronger continuity of spontaneous combustion.

The primary oxidation process, as depicted in Figs 36-43, reveals that among coal samples with the same minimum floating coal thickness, the maximal air leakage intensity is observed for the sample with an air flow of 50mL/min. This is followed by samples with air flows of 25mL/min and 100mL/min respectively, while the sample with an air flow of 200mL/min exhibits the highest minimum value. During secondary oxidation, under identical minimum floating coal thickness conditions, the smallest maximal air leakage intensity is found for the coal sample subjected to an experimental air flow of 100mL/min. Subsequently, samples with air flows of 25mL/min and 50mL/min display higher values successively; whereas a maximum minimum value is observed for the sample exposed to an air flow of 200mL/min. These results align consistently with variations in both minimum floating coal thickness and low limit oxygen flow fraction.

The same rules and conclusions can be derived through a comparative analysis of the primary and secondary oxidation processes.

In summary, the occurrence of spontaneous combustion in coal is more likely with larger air flows and is further facilitated by secondary oxidation. The primary oxidation has minimal influence on the limit parameters and judgment of spontaneous combustion for coal samples with high (200mL/min) and low (25mL/min) air flows. However, it significantly affects the limit parameters and judgment for coal samples with moderate air flows (50mL/min, 100mL/min). Compared to primary oxidation, secondary oxidation requires less coal, lower oxygen concentration, lower maximal air leakage intensity, exhibits a wider range of spontaneous combustion conditions, and demonstrates stronger persistence.

## 5 Conclusion

Under an air flow rate of 200 mL/min, the oxygen consumption rate and exothermic intensity of secondary oxidation in the coal samples exceed those of primary oxidation within the temperature range of 40–90°C. However, this trend undergoes a significant reversal when the temperature surpasses 90°C. In contrast, at air flow rates ranging from 25 to 100 mL/min, both oxygen consumption rate and exothermic intensity during secondary oxidation demonstrate a consistent elevation throughout the entire oxidation process. Notably, a reduction in air flow rate correlates with a diminished disparity between primary and secondary oxidation in terms of oxygen consumption rate and exothermic intensity.

At an air flow rate of 100 mL/min, the temperature at which CO2 exhibits significant growth during the secondary oxidation of coal samples is notably advanced compared to that observed during primary oxidation. Furthermore, upon reaching a critical temperature point of significant growth (120°C), the CO2 concentration during secondary oxidation surpasses that of primary oxidation. However, with subsequent increases in air flow, this critical temperature point for CO2 growth is significantly elevated, and the CO2 concentration during secondary oxidation becomes lower than that during primary oxidation.

Primary oxidation not only enhances the secondary oxidation process but also leads to increased CO production from oxidized coal. It reduces the apparent activation energy of coal in stages I and II, while in stage III, it decreases the apparent activation energy of coal samples at 200 mL/min and 50 mL/min, but increases the apparent activation energy at 100 mL/min and 25 mL/min. The likelihood of coal spontaneous combustion escalates with increasing air flow, and secondary oxidation further amplifies this risk.

Primary oxidation exerts a minimal impact on the assessment of spontaneous combustion risk for coal samples at 200 mL/min and 25 mL/min, but significantly influences the risk assessment for samples at 50 mL/min and 100 mL/min. The duration of spontaneous combustion induced by secondary oxidation is shorter than that caused by primary oxidation, thereby presenting a higher risk profile.

These findings underscore the complex interplay between air flow rate, oxidation processes, and spontaneous combustion risk, highlighting the need for nuanced risk assessment strategies in coal storage and handling environments.

## Supporting information

S1 Table This is the paper’s data. (XLSX)
